# Advanced Process Control for Clinker Rotary Kiln and Grate Cooler †

**DOI:** 10.3390/s23052805

**Published:** 2023-03-03

**Authors:** Silvia Maria Zanoli, Crescenzo Pepe, Giacomo Astolfi

**Affiliations:** 1 Dipartimento di Ingegneria dell’Informazione, Università Politecnica delle Marche, Via Brecce Bianche 12, 60131 Ancona, Italy; 2 Alperia Green Future, Via Dodiciville 8, 39100 Bolzano, Italy

**Keywords:** advanced process control, model predictive control, cement industry, clinker production, rotary kiln, grate cooler, fuel/coal specific consumption, electric energy consumption, energy-saving

## Abstract

The cement industry includes energy-intensive processes, e.g., clinker rotary kilns and clinker grate coolers. Clinker is obtained through chemical and physical reactions in a rotary kiln from raw meal; these reactions also involve combustion processes. The grate cooler is located downstream of the clinker rotary kiln with the purpose of suitably cooling the clinker. The clinker is cooled through the action of multiple cold air fan units as it is transported within the grate cooler. The present work describes a project where Advanced Process Control techniques are applied to a clinker rotary kiln and a clinker grate cooler. Model Predictive Control was selected as the main control strategy. Linear models with delays are obtained through ad hoc plant experiments and suitably included in the controllers’ formulation. A cooperation and coordination policy is introduced between the kiln and the cooler controllers. The main objectives of the controllers are to control the rotary kiln and grate cooler critical process variables while minimizing the fuel/coal specific consumption of the kiln and the electric energy consumption of the cold air fan units within the cooler. The overall control system was installed on the real plant, obtaining significant results in terms of service factor and control and energy-saving performances.

## 1. Introduction

The cement industry involves energy-intensive processes such as clinker rotary kilns and clinker grate coolers. Clinker is the fundamental component of the cement, which is produced through a baking process in a kiln and a subsequent cooling process in a cooler, and large amounts of thermal and electric energy are required [1]. In many cement industries, large energy efficiency improvement margins can be observed due to dated plant hardware and/or the not-optimized conduction of the subprocesses. In the last decade, Green Economy principles have gained wide acceptance: the cement industry has been involved in this transformation process and different studies were conducted in this context, elevating decarbonization as a topic of primary importance [2]. In this context, carbon dioxide (CO_2_) and pollutant emission reduction represents a significant objective in all cement industry subprocesses [3,4]. Clinker rotary kilns and grate coolers play an important role in terms of energy consumption and emissions, due to the huge amount of thermal and electric energy required [1].

Data selection, acquisition, storage and analysis retain a primary role in the design of decision support systems and expert systems [5] able to monitor, control and optimize the performance in the cement industry: Industry 4.0 technologies and digitalization can represent a strategic driver in this field [2,6]. Furthermore, for the mitigation of pollution, carbon dioxide (CO_2_) emissions and plant conduction costs, Advanced Process Control (APC) systems can be considered [7]. APC systems usually are located at the second level of the automation hierarchy [8], can be included in edge computing or cloud computing architectures [9] and are able to optimize in real-time the behavior of processes while taking into account different specifications of the critical process variables [10]. In addition, monitoring and high-level optimization solutions can play an important role with respect to other objectives, e.g., the accurate observation of critical process variables and the optimization of plant assets at a global level.

Different approaches for the monitoring, high-level optimization and APC of cement industry clinker rotary kilns and grate coolers have been proposed by researchers, engineers and practitioners. Monitoring and high-level optimization solutions are proposed in [11,12,13,14,15,16]. In [11], a review of the studies on emissions caused by cement complexes is reported, focusing on the treatment and on the monitoring of the pollution emissions. Modelization is here performed in Python and the objective is to assess how smart monitoring and modeling techniques can help on the road toward high-efficiency cement production and eco-friendly procedures. In [12], a process model of a precalciner kiln system in the cement industry using Aspen Plus software is proposed to simulate the effects of five alternative fuels on pollutant emissions and energy performance. The model is developed on the basis of the energy and mass balance of the system and is validated using data from a reference cement plant. A model for a cost-optimal cement plant that fulfils carbon limitations without compromising product specifications is designed in [13], focusing on three mitigation methods for cement manufacturing: co-processing, kiln system improvements, and carbon capture. The model was used for the formulation of a mixed integer linear programming problem. In [14], the performance of a grate cooler in a cement manufacturing industry in Nigeria has been examined using the mass and heat balance methodology. The performance evaluation parameters considered are clinker temperature, cooler loss and efficiency. This study revealed that the grate cooler efficiency estimated through heat consumption balances conformed to the designed standard. In [15], the nitrogen oxides (NO_x_) control problem is tackled. A data-driven model for the NO_x_ process variable is achieved and a real-time optimization problem is formulated and solved through a particle swarm optimization algorithm. In [16], a multi-objective formulation based on entropy generation is designed for clinker layer thickness control. A genetic algorithm is employed for the solution of the optimization problem, providing the minimum energy consumption of cooling fans.

The monitoring and high-level optimization algorithms reported in the previous paragraph are not in charge of the management of the real-time operation of the plants. In this context, APC systems can play a key role in rotary kiln and grate cooler control and optimization. APC system solutions for cement kilns are proposed in [17,18,19,20,21,22,23,24,25,26,27,28], while APC system solutions for coolers are proposed in [29,30,31,32,33,34,35,36].

With regard to kilns, in [17], mathematical models are exploited for the design of a conventional control system in order to control several variables in real-time. Proportional-Integral (PI) and Proportional-Integral-Derivative (PID) controllers are exploited, and MATLAB [37] software is used for simulation. In [18], an optimal control solution is proposed based on the Model Predictive Control (MPC) strategy: the weights of the controller are tuned using genetic algorithms and an interactive decision tree, taking into account the minimization of the tracking error and of the overall energy utilization; the proposed approach is validated through simulation scenarios. In [19], the clinker burner process is controlled and optimized through a first principles model. An MPC strategy is used to stabilize a temperature profile along the rotary kiln, while moving horizon estimation is used for the online estimation of selected model parameters and unmeasured states; the proposed control system was commissioned on a real plant. In [20], hybrid system theory, thermal regulation, and the least squares method are combined for the design of a simulation and control platform of rotary kiln burning zone temperature. In [21], an automatic control method of the burning temperature based on multi-control strategy combinatorial control technology is proposed. A set of combinatorial rules are formulated based on human operation knowledge and experience, and the proposed method was tested on a real plant. In [22], a data-driven autoregressive moving average model is established to predict the temperature of the flue gas chamber at the kiln tail. The model identification was based on standard goodness-of-fit metrics. The obtained model was used for the design of an MPC strategy that was tested through simulations. The control of the temperature of the burning zone of an industrial cement rotary kiln is addressed in [23]. A model with time delay is identified from experimental data and is used for the synthesis of different controllers, i.e., a standard PI controller, a PI controller with a Smith predictor scheme and a fractional-order controller with a Smith predictor scheme. The developed controllers are tested through simulations. In [24], a generalized predictive controller for the control of the clinkerization temperature of a cement rotary kiln is proposed. Real data are used for model identification and simulation scenarios are exploited to compare the developed controller with a PID controller. A state feedback Smith predictor controller for the temperature control in a precalciner of a cement rotary kiln is proposed in [25]. A dynamic model of the process is obtained by exploiting real industrial data; simulations are used for the comparison between the designed controller with a standard PID controller. In [26,27,28], a two-layer MPC strategy and slack variables formulation are used by the authors for the control and the optimization of a clinker rotary kiln without a precalciner. Cooperation policies between a Linear Programming (LP)-based optimizer (at the upper layer) and a Quadratic Programming (QP)-based optimizer (at the lower layer) are exploited. Different simulations based on constraints variation and on disturbances rejection allowed for the testing of the developed controllers, and finally, field implementation confirmed the validity of the proposed control approaches for clinker rotary kilns without precalciners.

With regard to clinker coolers, an optimization method for the performance of PID controllers is proposed in [29]. A process with a fixed grate and two moving ones is considered. A process model between the speed of the moving grate and the pressures of the static and of the moving grates is identified by industrial data. The process model is used for tuning and simulation purposes. The design of an internal model control-based PID controller for maintaining the under grate pressure of a grate cooler used in cement plants is presented in [30], with the goal of achieving target tracking for the considered controlled variable; the proposed approach is tested through simulations. In [31], the objectives of target tracking the chamber pressure of the grate cooler are analyzed while respecting a minimum constraint for the secondary air. A PI controller is designed based on a switching mechanism; the control strategy was tested in practical applications.

In [32], a multi-mode intelligent control method is proposed for the optimization of a grate cooler: variable integral PID control, fuzzy control, bang-bang control and expert control techniques are suitably combined and tested on industrial applications. In [33], the grate down pressure control and stabilization problems are addressed through an optimizer based on a least squares support vector machine; the proposed approach is tested through simulations. In [34], a dynamic matrix control strategy is exploited for grate cooler control, based on a prediction model obtained through the least-squares method; the proposed approach is tested through simulations. In [35], the expert system and fuzzy control system are applied to manipulate the grate speed in order to control the chamber pressure; the proposed approach is tested through field application. In [36], a back propagation algorithm in neural network modeling is applied to predict the grate chamber pressure and a fuzzy control technique is used; the proposed approach is tested through field application.

The present paper proposes the design and the field implementation of APC systems for the clinker rotary kiln and grate cooler of the cement industry. The paper aims to provide holistic knobs and solutions for the assessment of APC methods in the cement industry. The present paper extends the contents reported in [38], providing additional details and insights into the different phases of the developed project and emphasizing the provided novelties. To the best of the authors’ knowledge, in the literature of APC systems for clinker rotary kilns and grate coolers, the following technical aspects have not been explored in depth:

- The procedural steps for qualified plant survey and data selection, acquisition, storage and analysis phases play a fundamental role before beginning an APC project for cement plants. These phases have not been thoroughly detailed in the literature;
- An overall assessment of the Key Performance Indicators (KPIs) to be computed and exploited for the evaluation of the performance provided by an APC system on a grate cooler has not been thoroughly detailed in the literature.

In addition to the mentioned technical merits, the following innovative scientific contributions, not present in the literature, characterize the proposed paper:

- A procedure for the inclusion of the kiln filling degree in the control matrix of an APC system while maintaining a linear formulation for the process variables’ constraints;
- An architecture that allows the cooperation between different APC systems;
- An APC system scheme capable of taking into account bad data detection and local control loop malfunctions;
- A two-layer MPC scheme with a cooperation policy between the layers that can be characterized by LP or QP formulations. This feature allows the adaptation of an APC scheme to subprocesses with different control specifications;
- A method for the exploitation of sensor/analyzer redundancy in an APC system. This feature allows us to ensure a reliable control in all operating conditions, including tailored plant operations (e.g., cyclone cleaning);
- The definition of different priority rankings for controlled variable constraint relaxations based on the current operating conditions.

The control and energy-saving results achieved with the field implementation confirmed the validity of the proposed approach. To the best of the authors’ knowledge, projects that include implementation in the real process and are designed as lasting control applications and not as temporary tests are not widespread. The field application of an APC system designed and tested through virtual environment simulations requires significant reliability and robustness features in order to bridge the gap between simulations and field application.

The paper is organized as follows: Section 2 reports the material and methods, providing: the process description, the control specifications, and the project definition. In addition, some details on plant survey and on data selection, acquisition, storage, and analysis are reported. Finally, modelization, model mismatch compensation, APC design, and software details are described. Section 3 reports the results and discussion, focusing on data analysis, modelization, virtual environment simulations and field results. The conclusions are summarized in Section 4, together with some ideas for future work.

## 2. Materials and Methods

### 2.1. Process Description

**Figure 1:**
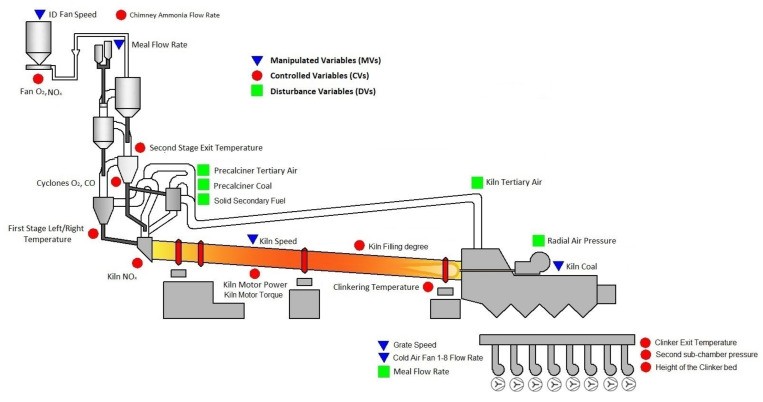
Clinker rotary kiln and grate cooler.

Figure 1 reports the considered process, represented by the clinker rotary kiln and grate cooler of an Italian cement industry. The clinker production phase at issue processes about 740,000 tons per year of raw meal, using about 64,000 tons per year of carbon coke. The average yearly consumption of carbon coke is about 47,000 tep.

Raw meal is suitably preheated in a suspension preheater composed of a cyclone tower (see the left side of Figure 1). Different cyclone stages characterize the tower, where a preheating/drying phase takes place. In the cyclone tower, raw meal is mixed with exhaust gas from downstream areas; an induced draft (ID) fan pulls up the air (see the left side of Figure 1). In each cyclone stage, separation and reunification between raw meal and exhaust gas takes place: the blending, separation and remixing phases are repeated until the material is discharged from the last cyclone stage to the rotary kiln. In the last stages of the cyclone tower, the precalcination phase takes place through the action of a precalciner located between the suspension preheater and the kiln. Here, a preliminary baking is performed through the combustion triggered with precalciner coal and solid secondary fuel. Here, tertiary air is exploited, which represents additional hot air achieved through heat recovery from the grate cooler [1].

The rotary kiln is a long, slightly sloped cylinder characterized by refractory material that is resistant to high temperatures. The kiln rotates around its axis, allowing the processed mixture to move along it. The kiln internal temperature can reach about 1600 °C near the clinkering zone (see the right side of Figure 1). Here, the main baking procedure takes place thanks to the action of a coal burner, and clinker is obtained. Subsequently, clinker is cooled in a grate cooler and then combined with other components, such as calcium sulfate or pozzuolan, obtaining the desired type of cement.

A typical temperature of clinker at the kiln exit is about 1200 °C. The next grinding phase requires temperatures of about 100 °C, so a cooling phase is performed in the grate cooler (see the right side of Figure 1). The grate cooler, in addition to the previously mentioned tertiary air, also recovers the clinker excess heat and supplies it to the combustion air; the achieved hot air is referred to as secondary air. The cooling is performed through the action of cold air fan units: the provided cold air is conveyed through some overlapping perforated plates. These plates move alternately in order to transport the clinker. The grate cooler considered consists of eight sub-chambers. The pressure of the sub-chambers must provide air pressure that can penetrate the bed of hot clinker of a given thickness and permeability [39].

#### 2.1.1. Sensors/Analyzers Equipment and Process Automation

In the considered process, different sensors/analyzers and laboratory analysis are available in order to monitor the current conditions. For example, temperatures are measured by suitable sensors in the cyclone stages and within the kiln (see the left side and right side of Figure 1) and by an optical pyrometer in the last part of the grate cooler (see the right side of Figure 1). Suitable analyzers measure the carbon monoxide (CO) concentration and the oxygen (O_2_) and nitrogen oxides (NO_x_) levels near the ID fan (see the top left side of Figure 1), in the cyclone stages (see the left side of Figure 1) and at the kiln inlet (see the bottom left side of Figure 1). The sensor related to the height of the clinker bed within the grate cooler (see the bottom right side of Figure 1) was installed after the commissioning of the APC system, and it was included in the APC design.

With regard to the process automation, local PID controllers manage the control loops of the ID fan speed, meal flow rate, precalciner tertiary air, precalciner coal, solid secondary fuel, kiln speed, kiln tertiary air, radial air pressure, kiln coal, grate speed and cold air fans [40].

In addition, free lime is monitored through the laboratory analysis of clinker samples. These samples are collected at the end of the rotary kiln and the analysis is performed four times per day. Free lime values have to lie in the range 0.8–1.2%.

#### 2.1.2. Control Specifications and Project Definition

With regard to the rotary kiln, the required environmental, thermodynamic, mechanical and quality constraints had to be respected in order to ensure the proper triggering of the involved chemical and physical reactions. The main control and optimization specifications in a clinker production unit of a cement plant are:

- Ensure proper combustion control;
- Take into account the clinker quality and the compounds quality;
- Reduce the energy consumption;
- Guarantee stabilized process conduction;
- These specifications can be targeted respecting the previously mentioned constraints:
- Environmental constraints: relate to carbon monoxide concentration, nitrogen oxides levels and chimney ammonia flow rate;
- Thermodynamic constraints: related to cyclones and kiln zones temperatures, as well as oxygen concentration;
- Mechanical constraints: involve kiln motor power, kiln motor torque and kiln filling degree;
- Quality constraints: related to free lime analysis (see Section 2.1.1).

Additional control specifications were established together with the process technologists, which address the limitations of the use of manipulated variables to control certain process variables. For example, the following specifications were required:

- The meal flow rate should not be used for kiln filling degree control;
- The ID fan speed should not be used for kiln nitrogen oxides control.

Meeting the described constraints and specifications, optimal operating points have to be ensured that minimize fuel/coal specific consumption, thus monitoring energy-saving and pollution impact aspects.

With regard to the grate cooler, the clinker bed has to be adjusted in response to the changes in raw meal flow rate, thereby eliminating the red river or blown-through phenomenon. The suitable control of the pressure of the second sub-chamber and of the height of the clinker bed is needed, while ensuring the quality of the clinker cooling by observing the optical pyrometer measurements (see Section 2.1.1). In this context, the electric energy consumption of the cold air fan units has to be minimized while guaranteeing the necessary heat recovery to the kiln and to the suspension preheater (see Figure 1).

The overall process control has to be ensured under all process conditions, including, for example, tailored plant operations (e.g., cyclone cleaning) and unexpected events (e.g., malfunction of a device).

Before the installation of the proposed APC systems, the control of the clinker rotary kiln and grate cooler was managed by plant operators manually and/or through standard control logics. Plant operators manually adjusted the setpoints of the ID fan speed, meal flow rate, precalciner coal, solid secondary fuel, kiln speed and kiln coal; in addition to manual control, standard control logics could intervene on grate speed and on cold air fan unit flow rates.

Thanks to an accurate plant survey and to an accurate data analysis (see Section 2.2), some control and optimization margins were detected in the considered processes. The large inertia, pure hysteresis, nonlinearity, nonstationary behavior and strong coupling characteristics, which affect the kiln and the cooler processes, motivated the proposal of an APC project exploiting MPC as a control and optimization technique.

**Figure 2:**
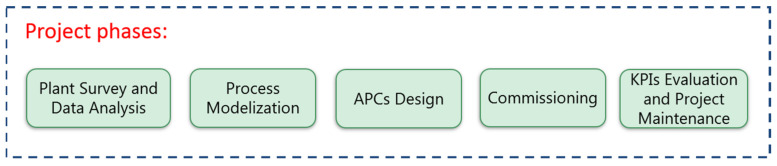
Project phases.

Figure 2 reports the main project phases. The process modelization phase (see Section 2.4) was consequent to the initial plant survey and data analysis phase; subsequently, the design of the APC systems (see Section 2.5) and the commissioning were conducted. Finally, KPI evaluation (see Section 2.3) and project maintenance phases represented the conclusion of the project phases. The accurate definition of the inputs and outputs to be obtained in each project phase represented a critical step.

Thanks to the nature of the considered processes, two APC systems were designed and a cooperation policy between them was established (see Section 2.5). The first APC system is responsible for rotary kiln control while the second one is in charge of the grate cooler control.

The list of the Manipulated Variables (MVs), Controlled Variables (CVs) and measured Disturbance Variables (DVs) [41] of each APC system is reported in Table 1, Table 2, Table 3, Table 4, Table 5 and Table 6. In Figure 1, the positions of the variables are shown schematically.

### 2.2. Plant Survey and Data Selection, Acquisition and Storage

The plant survey was a fundamental step for the APC project. Thanks to the plant survey, tailored interviews were performed with plant operators, technologists and engineers.

The selection of the process variables to be acquired and stored (reported in the previous section), together with the definition of the hardware and software architecture to be exploited in real-time, was a key phase that required special effort. In addition, free lime analysis was selected together with the variables responsible for energy consumption measurement, e.g., electric energy.

**Figure 3:**
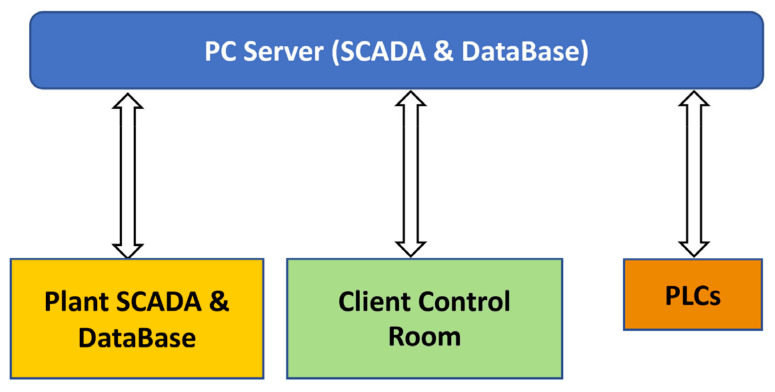
Data acquisition and storage architecture.

Figure 3 shows the architecture designed for acquisition and storage of the selected data. A PC server was installed in the plant, and it was connected to the plant network infrastructure (*PC Server* (*SCADA and Database*)). A Supervisory Control And Data Acquisition (SCADA) system was installed, and a database was created on the PC server. The PC server acquired data from a SCADA system, from a database already present in the plant (*Plant SCADA and Database*), and from plant Programmable Logic Controllers (*PLCs*). In particular, free lime analysis results are entered by plant operators on the plant SCADA system and stored in the plant database.

### 2.3. Data Analysis

Subsequent to the plant survey and to data selection, acquisition and storage, described in the previous section, a tailored data analysis procedure was designed and implemented [42]. The data analysis phase was divided into four main subphases:

- Process variables and setpoints analysis and processing;
- Local control loops performance evaluation;
- Detection, reporting and analysis of particular process conditions;
- KPI definition.

The process variables and setpoints analysis and processing subphase involved the process variables being measured by sensors/analyzers, free lime laboratory analysis and the setpoints commanded to the local controllers. First, measurement/acquisition errors/malfunctions were investigated, together with PLCs communications issues. Suitable data preprocessing techniques (e.g., validity limits and spike and freezing checks) were applied in order to detect the bad data to be discarded/replaced. In addition, suitable exponential filters were used to improve the robustness of selected measurements. The applied filters have the form:

(1a) $$m_{f}\left(k\right)=\alpha \cdot m_{f}\left(k-1\right)+\left(1-\alpha \right)\cdot m\left(k\right)$$

(1b) $$\alpha =e^{-\delta };\;\;\;\;\delta =\frac{T_{s}}{\tau }$$

where k is the discrete-time instant, $m\left(k\right)$ is the measurement at instant k and $m_{f}\left(\cdot \right)$ is the filtered measurement. $T_{s}$ is the sampling time and τ is the time constant of the exponential filter, both measured in seconds.

The local control loops performance evaluation subphase consisted of an assessment of the performances of the local controllers of MVs and DVs. Experimental tests were performed, consisting of suitable step moves on the setpoints, evaluating the rise time, overshoot and settling time. Furthermore, deviation conditions between setpoints and process variables were investigated [41].

Through data analysis, particular known process conditions were examined; one particular condition was cyclone cleaning, which is a standard procedure that is periodically executed on the suspension preheater. Bad detection logics were developed so to automatically detect sensor unreliability. Sensor unreliability can be automatically detected through the bad detection logics; in addition, plant operators, through the SCADA interface (see Section 2.5.2), could specify known anomalous operation conditions.

The previously mentioned data analysis procedures were customized in order to be implemented in the real-time APC systems through a module named *Bad Detection, Data Conditioning, Decoupling Selector and Model Selector*. An overall data analysis reliability flag results from the data analysis checks for the kiln and for the cooler. In addition, checks are suitably grouped to obtain critical conditions, for example, the unavailability of all oxygen measurements. This flag is exploited by the APC systems (see Section 2.5). Based on the mentioned checks, a dynamic adjustment of the control matrix is performed by the *Bad Detection, Data Conditioning, Decoupling Selector and Model Selector* module (see Section 2.5) through suitable status values and a decoupling matrix [10,41,43]. In this way, the inclusion of the MPC variables is suitably adapted online.

Data analysis also allowed us to define the main KPIs to consider for APC system performance evaluation. With regard to the kiln, the following KPIs were selected:

- Mean value and standard deviation of the cyclone oxygen;
- Mean value and standard deviation of the kiln nitrogen oxides;
- Kiln average fuel/coal specific consumption [3,4];
- Service factor, i.e., the percentage of time the APC system is fully in service.

The kiln fuel/coal average specific consumption is computed, taking into account different terms, e.g., the meal flow rate, the kiln coal, the precalciner coal and the solid secondary fuel. With regard to the grate cooler, the following KPIs were selected:

- Mean value of the temperature of the kiln tertiary air; this process variable provides an indication of the grate cooler conduction stability and uniformity of the clinker distribution;
- Mean value and standard deviation of the second sub-chamber pressure;
- A counter related to the second sub-chamber pressure that measures the times that this process variable exceeds a maximum pressure threshold (equal to 73 mbar);
- Cooler electric energy consumption;
- Service factor, i.e., the percentage of time the APC system is fully in service.

It was not possible to use as a KPI the height of the clinker bed because the sensor was installed after the commissioning of the APC system (see Section 2.1.1).

The definition of the KPIs allows us to compare the system performances pre and post commissioning the APC system, providing some metrics to prove robustness and reliability as well.

To the best of the authors’ knowledge, the proposed methods for plant surveying, data selection, acquisition, storage and analysis represent a technical innovation for APC systems on cement plants, with a specific focus on clinker rotary kilns and grate coolers. In addition, to the best of the authors’ knowledge, the grate cooler control KPI represented by the counter on the second sub-chamber pressure represents an additional technical innovation in the literature of APC systems on cement-industry clinker grate coolers. Not executing an in-depth plant survey and/or not using accurate methods of data selection, acquisition, storage and analysis may mean missing a key prerequisite for designing robust and lasting APC systems. In addition, a non-accurate choice of the KPIs to be observed for the evaluation of the installation of an APC system could cause an incomplete assessment of the overall benefits provided.

### 2.4. Modelization and Model Mismatch Compensation

In order to design an MPC solution for the processes under consideration, the modelization is a fundamental requirement because MPC techniques strictly depend on the obtained process model [44]. A linear modelization approach, based on empirical data-based models [45] and first principles equations [1], was adopted for both the rotary kiln and the grate cooler.

A data-based modeling approach was adopted for the related relationships of all MVs-CVs and DVs-CVs pairs (see Table 1, Table 2 and Table 3 for the kiln and Table 4, Table 5 and Table 6 for the cooler), excluding the pairs that involve the kiln filling degree (see Table 3), the second sub-chamber pressure and the height of the clinker bed (see Table 6 and Section 2.5.3). All CVs (excluding the kiln filling degree) are included in suitable y vectors for the kiln and for the cooler; similarly, MVs/DVs are included in suitable u/d vectors. An ad hoc data collection phase was designed in order to capture the most significant dynamics, and step test procedures were executed on the processes at different conditions, suitably acting on inputs (MVs, DVs). The different conditions refer to different values of the meal flow rate; in particular, two conditions were considered: “low-medium meal flow rate” and “high meal flow rate”. A different process model was identified for each operating condition. Suitable hysteresis logics were introduced in order to avoid chattering on the process model selection and to properly handle the transients between the different operating conditions.

For exploiting the step test data for each input-output pair (i.e., for each MV-CV and DV-CV pair), a First Order Plus Dead Time (FOPDT) model (asymptotically stable) was adopted, and suitable parameters’ values were identified [45]. Considering a multi-input multi-output (MIMO) transfer function matrix, a discrete time state-space realization was finally computed for the kiln and for the grate cooler [46]. In accordance with the obtained process models and the computational load required by the overall control algorithm, a sample time of 60 s (one minute) was selected for the kiln APC system, while a sample time of 30 s was selected for the cooler APC system. In order to face model uncertainties and sensor noise/errors, additional state and unmeasured disturbance variable terms were included in the MPC linear models [47,48]. A tailored state estimation problem was solved through a deadbeat Kalman filter [49,50].

As previously mentioned, the input–output relationships related to kiln filling degree (kiln CV13, see Table 3) were not modelized through empirical models (a sensor was not available), and the following first principles discrete-time equation was formulated:

(2) $$CV13\left(k\right)=c\left(\rho \right)\cdot \frac{MV1\left(k\right)}{MV3\left(k\right)}$$

In (2), k is the discrete-time instant. The kiln filling percentage degree is a nonlinear function of the meal flow rate, in t/h (kiln MV1, see Table 1), and of the rotation kiln speed, in rpm (kiln MV3, see Table 1). c, in (h·rpm·%)/t, takes into account the kiln slope, length, inner diameter and the raw meal density ρ. In order to avoid a nonlinear kiln MPC formulation, and assuming that the kiln APC system cannot be active if the kiln is not rotating, Equation (2) is reformulated as in the linear Equation (3):

(3) $$z\left(k,\rho \right)=CV13_{t}\left(k\right)\cdot MV3\left(k\right)-c\left(\rho \right)\cdot MV1\left(k\right)=0$$

where z represents a fictitious variable and $CV13_{t}$ represents the current desired value for the kiln filling degree. As will be explained in Section 2.5.2, the z variable is included in the kiln MPC formulation, and its constraints are set equal to zero.

To the best of the authors’ knowledge, the formulated Equation (3) represents a scientific contribution to the literature of clinker rotary kilns APC systems.

### 2.5. APCs Design

As schematized in Figure 2, the APC design phase is executed after the plant survey, data analysis and modelization steps. In order to comply with the defined specifications (see Section 2.1.2), MPC was selected as the control strategy [51,52]. As mentioned in the previous sections, two APC systems were designed and a cooperation policy between them was established.

**Figure 4:**
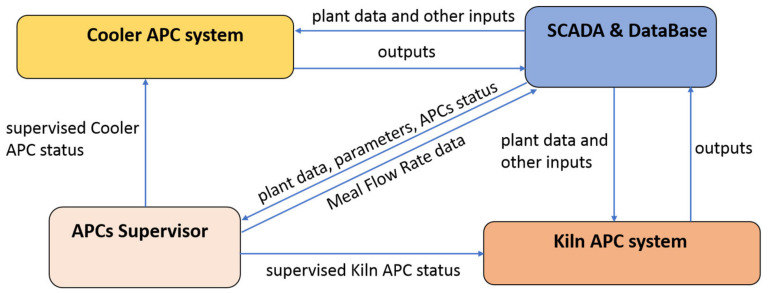
APCs cooperation architecture.

Figure 4 shows the APC systems and the architecture designed for their cooperation. The SCADA and database of the PC server block described for Figure 3 can be noted also in Figure 4: it provides data and parameters to the other modules. An APCs supervisor module (*APCs Supervisor*) was designed in order to implement a cooperation policy between the kiln APC system (*Kiln APC System*) and the cooler APC system (*Cooler APC System*). This module runs with the cooler APC system sampling time (30 s) and carries out two tasks:

1. Definition of a supervised APC status value for the both the kiln and cooler APC system;
2. Definition of the meal flow rate data to be used in the cooler APC system.

The supervised APC status values for the kiln/cooler APC system take into account the APCs status value (see in the following) and a criticality matrix, which defines whether an APC system can be active while the other APC system is not active. In addition, the APCs supervisor module defines the meal flow rate data to be used in the cooler APC system. Meal flow rate is a measured DV for the cooler APC system (see Table 5), while it is an MV for the kiln APC system (see Table 1). At each control instant (at each minute), if active, the kiln APC system computes a sequence of meal flow rate values on the defined kiln control horizon ($H_{u}$) [53]. These data are conditioned by the APCs supervisor module in order to provide the meal flow rate data to the cooler APC system. Through the developed cooperation policy, the clinker bed in the grate cooler is adjusted in response to the changes in raw meal flow rate of the kiln; in this way, a coordination between the kiln control and the cooler control is obtained.

To the best of the authors’ knowledge, the proposed cooperation architecture of Figure 4 represents a scientific contribution to the literature of clinker rotary kilns and grate coolers APC systems.

**Figure 5:**
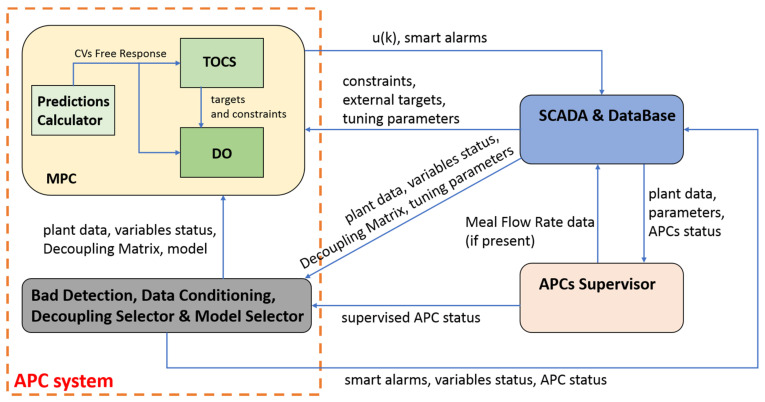
Details on the modules of the APC systems.

Additional details on the modules of the APC systems of Figure 4 are reported in Figure 5. The concepts already stated for the description of Figure 4 hold. The *SCADA and Database* module (also see Section 2.2 for further details on this module) provides plant data and parameters to all the other modules at each control instant k. In addition, the *SCADA and Database* module provides an initial APC status flag (Figure 5, *APCs status*) to the *APCs Supervisor* module. This flag defines if the considered APC system can conduct the plant. For example, if a watchdog communication error is detected in the communication between the SCADA and the PLCs (see Figure 3), the APC system conduction is disabled.

The APC status flag provided by the *SCADA and Database* module is processed by the *APCs Supervisor* module in order to compute the final supervised APC status flag to be used in each APC system (see Figure 4 and Figure 5). This flag, combined with the *data analysis reliability flag* described in Section 2.3, contributes to the computation of the APC status flag that is returned to the *SCADA and Database* module by the *Bad Detection, Data Conditioning, Decoupling Selector and Model Selector* module (see Figure 5), together with smart alarms. Furthermore, this module uses bad detection results (see Section 2.3) and the final APC status value, together with the process variables status value provided by the *SCADA and Database* module [41,43], in order to compute the final status value for each process variable (see *variable status* term on the bottom left of Figure 5). This value determines if and how the considered process variable will be included in the MPC problem at the current control instant.

The *Bad Detection, Data Conditioning, Decoupling Selector and Model Selector* module, together with the *SCADA and Database* module, provides the model, data and parameters to the *MPC* module that, exploiting a receding horizon strategy (see in the following), computes the MVs value to be applied to the plant (Figure 5, $u\left(k\right)$).

To the best of the authors’ knowledge, the proposed APC architecture of Figure 5 represents a scientific contribution to the literature of clinker rotary kilns and grate coolers APC systems.

#### 2.5.1. MPC Module

The *MPC* module of each APC system (see Figure 5) is composed by three blocks: *Predictions Calculator*, *Targets Optimizing and Constraints Softening* (*TOCS*) and *Dynamic Optimizer* (*DO*). The *Predictions Calculator* block, based on the current linear process model, computes the CVs *free response* [53] over the prediction horizon ($H_{p}$), i.e., the CVs predictions when no future moves are performed on the MVs. The MVs future control moves are indicated with $\Delta \hat{u}\left(k+i|k\right)$ terms ($i=0,\ldots ,\;H_{u}-1$); at each control instant k, only the first move computed by the *MPC* module is used to compute the MVs value to be sent to the plant.

The other two blocks of the *MPC* module implement a two-layer optimization strategy: the *TOCS* module represents the upper layer, while the *DO* module constitutes the lower layer. The MVs sequence is computed by *DO* module through a QP problem, while *TOCS* module computes feasible and consistent constraints and targets steady-state configuration for the *DO* module (see in the following) based on a LP [41] or QP problem.

The generic *DO* module QP formulation minimizes the following quadratic cost function:

(4) $$V\left(k\right)=\overset{H_{u}-1}{\underset{i=0}{\sum }}∥\Delta \hat{u}\left(k+i|k\right){∥}_{R\left(i\right)}^{2}+\overset{H_{u}-1}{\underset{j=0}{\sum }}∥\hat{u}\left(k+i|k\right)-u_{t}\left(k+i|k\right){∥}_{S\left(i\right)}^{2}+∥\varepsilon {\left(k\right)∥}_{\rho }^{2}$$

subject to the linear constraints:

(5a) $$lb_{du}\left(i\right)\leq \Delta \hat{u}\left(k+i|k\right)\leq ub_{du}\left(i\right),\;\;\;\;\;i=0,\;\ldots ,\;H_{u}-1$$

(5b) $$lb_{u}\left(i\right)\leq \hat{u}\left(k+i|k\right)\leq ub_{u}\left(i\right),\;\;\;\;i=0,\;\ldots ,\;H_{u}-1$$

(5c) $$lb_{y}{}_{j}\left(k+i\right)-\gamma_{lb,y}{}_{j}\cdot \varepsilon \left(k\right)\leq {\hat{y}}_{j}\left(k+i|k\right)\leq ub_{y}{}_{j}\left(k+i\right)+\gamma_{ub,y}{}_{j}\cdot \varepsilon \left(k\right),\;\;\;\;\;\;\;j=1,\;\ldots ,m_{y};\;\;\;i=H_{w}{}_{j},\ldots ,\;H_{p}$$

(5d) $$lb_{z}{}_{j}\left(k+i\right)-\gamma_{lb,z}{}_{j}\cdot \varepsilon \left(k\right)\leq {\hat{z}}_{j}\left(k+i|k\right)\leq ub_{z}{}_{j}\left(k+i\right)+\gamma_{ub,z}{}_{j}\cdot \varepsilon \left(k\right),\;\;\;\;\;\;\;j=1,\;\ldots ,m_{z};\;\;\;i=0,\ldots ,\;H_{p}$$

(5e) $$\varepsilon \left(k\right)\geq 0$$

The generic *TOCS* module QP (or LP) formulation minimizes the following quadratic (or linear) cost function:

(6) $$V_{TOCS}\left(k\right)=c_{u}{}^{T}\cdot \Delta {\hat{u}}_{TOCS}\left(k\right)+∥\Delta {\hat{u}}_{TOCS}\left(k\right){∥}_{R_{TOCS}}^{2}+∥{\hat{u}}_{TOCS}\left(k\right)-u_{t}{}_{TOCS}\left(k\right){∥}_{S_{TOCS}}^{2}+f_{TOCS}^{T}\cdot \varepsilon_{TOCS}\left(k\right)+∥\varepsilon_{TOCS}\left(k\right){∥}_{\rho_{TOCS}}^{2}$$

subject to the linear constraints:

(7a) $$lb_{du\_TOCS}\leq \Delta {\hat{u}}_{TOCS}\left(k\right)\leq ub_{du\_TOCS}$$

(7b) $$lb_{u\_TOCS}\leq {\hat{u}}_{TOCS}\left(k\right)\leq ub_{u\_TOCS}$$

(7c) $$lb_{y\_TOCS}-\gamma_{lby\_TOCS}\;\cdot \;\varepsilon_{TOCS}\left(k\right)\leq {\hat{y}}_{TOCS}\left(k\right)\leq ub_{y\_TOCS}+\gamma_{uby\_TOCS}\;\cdot \;\varepsilon_{TOCS}\left(k\right)$$

(7d) $$lb_{z\_TOCS}-\gamma_{lbz\_TOCS}\;\cdot \;\varepsilon_{TOCS}\left(k\right)\leq {\hat{z}}_{TOCS}\left(k\right)\leq ub_{z\_TOCS}+\gamma_{ubz\_TOCS}\;\cdot \;\varepsilon_{TOCS}\left(k\right)$$

(7e) $$\varepsilon_{TOCS}\left(k\right)\geq 0$$

In Equations (4) and (6), ∥·∥ represents the Euclidean norm while the superscript T indicates the transpose operator. The formulated LP optimization problems are solved through the active-set algorithm while QP optimization problems are solved through the interior point algorithm [53].

*DO* and *TOCS* modules, as stated before, take into account process variables predictions over the $H_{p}$. MVs and CVs predictions over $H_{p}$ are considered by the *DO* module ($\hat{u}\left(k+i|k\right)$ and ${\hat{y}}_{j}\left(k+i|k\right)$/${\hat{z}}_{j}\left(k+i|k\right)$ in Equations (4) and (5b–d)), while the predictions at $H_{p}$ (steady-state) are included in the *TOCS* formulation (${\hat{u}}_{TOCS}\left(k\right)$ and ${\hat{y}}_{TOCS}\left(k\right)$/${\hat{z}}_{TOCS}\left(k\right)$ in Equations (6) and (7b–d). In (5c), $H_{w}{}_{j}$ represents the first prediction instant where the associated CV can be constrained: its definition in the proposed formulation is based on the time delays values of the FOPDT models (see Section 2.4). $\Delta \hat{u}\left(k+i|k\right)$ terms in Equations (4) and (5a) indicate the *DO* MVs future control moves (assumed here in the first consecutive $H_{u}$ prediction steps [54]), while $\Delta {\hat{u}}_{TOCS}\left(k\right)$ represents the *TOCS* MVs move in Equations (6) and (7a).

Both the *DO* and *TOCS* modules include process variables constraints (Equation (5a–e) and Equations (7a–e)); MVs constraints (Equations (5a,b) and (7a,b)) are considered as inviolable (*hard*) constraints, while CVs constraints (Equations (5c,d) and (7c,d)) are set as *soft* constraints [44,53]. All constraints can be modified in real-time through the developed SCADA system (see in the following). MVs constraints refer to magnitude constraints (Equations (5a) and (7a)) and moves constraints (Equations (5b) and (7b)). Thanks to the data analysis described in Section 2.3, moves constraints are set taking into account local control loops behavior. Within the *MPC* module, suitable logics for the preprocessing of modified MVs magnitude constraints were implemented in order to always preserve the feasibility of the optimization problems to be solved.

CVs *soft* constraints can be relaxed, if needed, through the action of suitable nonnegative slack variables included in $\varepsilon \left(k\right)$ and $\varepsilon_{TOCS}\left(k\right)$ vectors (see Equations (4)–(7e)). The slack variables were introduced in the linear constraints together with suitable positive coefficients γ and are introduced in the cost functions through suitable matrices (ρ, $\rho_{TOCS}$) or vectors ($f_{TOCS}$).

For the solution of the optimization problems reported in (4)–(7e), the decision variables of the *DO* module are the $\Delta \hat{u}$ and ε terms, while the decision variables of the *TOCS* module are the $\Delta {\hat{u}}_{TOCS}$ and $\varepsilon_{TOCS}$ terms.

Move suppression terms can be included in the *TOCS* formulation through the $R_{TOCS}$ matrix, while they are always present in the *DO* formulation through $R\left(i\right)$ matrices. *TOCS* formulation includes the option of penalizing the magnitude of the MVs move in Equation (6), through suitable coefficients contained in the $c_{u}$ vector. In addition, MVs external targets can be supplied by the *SCADA and Database* module (see Figure 5 and $u_{t}{}_{TOCS}\left(k\right)$ in Equation (6)): the penalization of the tracking error is formulated in Equation (6) through suitable weights contained in $S_{TOCS}$ matrix. The *TOCS* module, solving its optimization problem, computes a feasible and consistent process configuration at $H_{p}$ (steady-state): this asset is represented by the terms ${\hat{u}}_{TOCS}\left(k\right)$, ${\hat{y}}_{TOCS}\left(k\right)$, and ${\hat{z}}_{TOCS}\left(k\right)$ (see Equations (6) and (7b–d)). These terms are provided as targets to the *DO* module, and they are used for the calculation of the MVs reference trajectories $u_{t}\left(k+i|k\right)$ (see Equation (4)). The tracking error with respect to the MVs reference trajectories is penalized in Equation (4) through suitable weights included in the $S\left(i\right)$ matrices [41].

To the best of the authors’ knowledge, the proposed two-layer MPC scheme with the described cooperation policy, characterized by the option of selecting the LP or QP formulation for the upper layer, represents a scientific contribution to the literature of clinker rotary kilns and grate coolers APC systems. This feature represents a holistic mathematical approach that allows the entire process to be controlled through the same general scheme.

#### 2.5.2. Kiln APC System Details

With regard to the kiln APC system, the suitable customization of the modules presented in the previous section was conducted. As previously mentioned, a sample time of 60 [s] (one minute) was selected for the kiln APC system. Furthermore, a prediction horizon equal to 150 prediction instants (150 min) was set in order to capture the significant part of the behavior of all CVs based on the two obtained process models (see Figure 5 and Section 2.3); the control horizon was set equal to 20 control moves [55].

**Figure 6:**
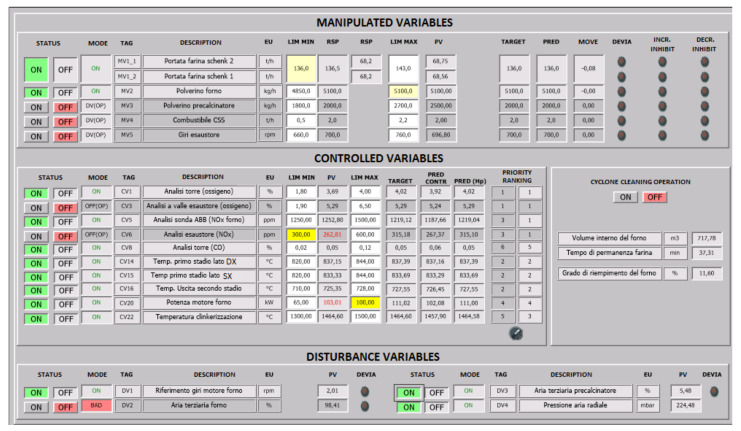
Rotary kiln APC system: example of an APC system GUI synoptic (MVs, main CVs and DVs).

As discussed in Section 2.3, through the developed real-time data analysis procedures, the presence of unexpected events or of particular plant operations, such as, for example, cyclone cleaning, can be detected. In this way, the unavailability, in practice, of certain variables’ measurements is detected. In addition, the operators can manually notify the start and the end of the cyclone cleaning operation (see the button on the right of Figure 6). In order to guarantee the applicability of the APC system in these situations, a redundancy of process variables in tailored plant operations (e.g., cyclone cleaning) and unexpected events (e.g., malfunction of a device) was developed and implemented through the SCADA system. Redundant CVs were considered in the APC system and suitably grouped, as shown in Table 7. One or more CVs of the same group can be active at each control instant, but the automatic redundancy method aims at defining rules so to ensure, when possible, that at least one CV of each group is included in the control problem (through the process variables status values, see Figure 5). Suitable criticality matrices were defined in order to correctly define the control matrix to be exploited at each control instant and to deduce if the kiln APC system could remain active; the information about the temporary elimination of a process variable by the control problem is included in the status values defined in the previous section (see Figure 5). The status values associated with the kiln process variables can be observed in Figure 6, where an example of the designed Graphical User Interface (GUI) of the kiln APC system is reported.

Examples of defined rules resulting from the data analysis regard oxygen (group 1 in Table 7) and nitrogen oxides (group 2 in Table 7). Cyclone oxygen is the key oxygen concentration to be kept under control, but its analyzer can clog generating drifts in the process variables value. In this condition, the cyclone oxygen is no longer controlled but the concentration of another oxygen (e.g., fan oxygen) is controlled in its place. A similar logic can be applied to kiln nitrogen oxides that represent the main NO_x_ variable; this variable must be replaced by chimney ammonia flow rate during cyclone cleaning. In addition, if a malfunction of the kiln nitrogen oxides analyzer occurs during normal production, fan nitrogen oxides is controlled instead.

In order to comply with the specifications described in Section 2.1.2 that require not using some MVs for the control of some CVs, an initial decoupling matrix (see Figure 5) is provided by the *SCADA and Database* module [10]. This matrix is reported in Table 8. The generic $\left(i,j\right)$ element of the decoupling matrix refers to the relationship between the ith CV of Table 3 and the jth MV of Table 1. In particular, the zero elements represent the relationships to inhibit according to the mentioned specifications. For example, for the third CV of Table 3 (kiln nitrogen oxides), ID fan speed must not perform any control action; this specification is represented by the zero element on the position (3,4) of the initial decoupling matrix reported in Table 8. The initial decoupling matrix provided by the *SCADA and Database* module is successively processed by the *Bad Detection, Data Conditioning, Decoupling Selector and Model Selector* module (see Figure 5). Eventually, the decoupling matrix is modified by this module, adding the information about the temporary elimination of a process variable (MV, CV) by the control problem. In this way, a control matrix dynamic adjustment is achieved. The final decoupling matrix is suitably introduced in the *TOCS* and *DO* blocks formulations [10,41].

As mentioned in Section 2.4 (see Equation (3)), a fictitious variable z was introduced in order to control the kiln filling degree. The obtained Equation (3) represents a linear equality constraint that was suitably introduced in Equations (5d) and (7d), only in the kiln APC system.

The *TOCS* module of the kiln APC system is characterized by an LP formulation, which implies that the quadratic terms reported in Equation (6) are not present for this controller. The remaining terms allow us to search the minimization directions for the fuel/coal specific consumption of the kiln while respecting process constraints; for this purpose, the element of the vector $c_{u}$ associated with the meal flow rate is set as negative (in order to maximize this MV when possible), while the element associated with the coal is set as positive (in order to minimize this MV when possible); the other elements were set equal to zero.

Within the *DO* module of the kiln APC system, a trade-off between the minimization of the magnitude of the control moves and the minimization of the tracking error with respect to the defined MVs targets was ensured in the quadratic cost function (see Equation (4)), through the suitable tuning of the positive definite matrices $R\left(i\right)$ and$\;S\left(i\right)$. However, this objective was set with a lower priority with respect to *soft* constraints satisfaction (see Equation (5c,d)) by suitably tuning the positive definite matrix ρ (see Equation (4)).

A grouping policy for the CVs constraints relaxations was formulated in the *DO* module, in order to limit the computational effort required for the solution of the optimization problem. All the CVs of the same group (see Table 7) share the same slack variable in the *DO* formulation; for example, cyclone oxygen and fan oxygen share the same slack variable. In addition, in order to guarantee the best tuning in all operating conditions, two priority rankings among the different groups of Table 7 were designed (see Table 9). One of the differences between the two priority rankings of Table 9 is represented by the rank assigned to the nitrogen oxides (group 2 in Table 7). Since this variable is not considered a crucial variable in all process conditions, its priority may require changing online, according to plant operation. The priority rank selector can be observed in Figure 6.

Within the kiln APC system, the sporadic feedback from free lime analysis (see Section 2.1.1) is taken into account through a correction of coal and meal flow rate constraints, based on the desired free lime target.

To the best of the authors’ knowledge, the proposed method for the exploitation of the sensor/analyzer redundancy (associated with some CVs) represents a scientific contribution to the literature of clinker rotary kilns APC systems. This feature makes it possible to ensure the best control actions in all operating conditions and to also use the APC system in the case of tailored plant operations (e.g., cyclone cleaning) and unexpected events (e.g., malfunction of a device). In addition, to the best of the authors’ knowledge, the proposed priority rankings for CVs constraints relaxations represent a further scientific contribution to the literature of clinker rotary kilns APC systems; a fixed tuning could not ensure the desired performance in all operating conditions, and for this reason switching between different tuning settings represents an important feature.

#### 2.5.3. Cooler APC System Details

**Figure 7:**
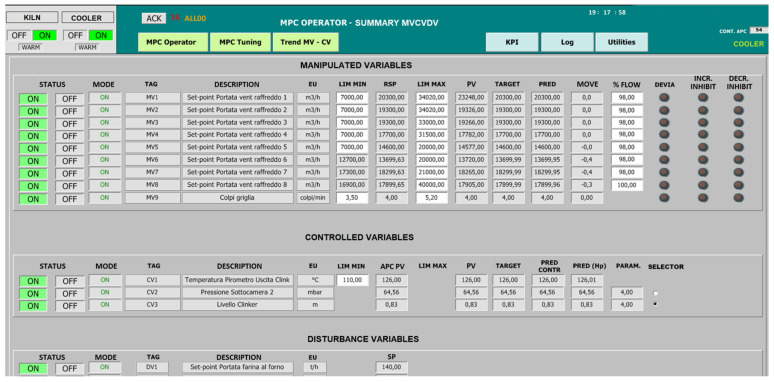
Grate cooler APC system: example of process variables GUI synoptic.

With regard to the cooler APC system, the suitable customization of the modules described in the previous section (see Figure 5) was conducted. Similar to what was performed for the kiln, different groups for the CVs were defined for the cooler (see Table 10 and Figure 7). In general, it is necessary for all CVs to be active to ensure fully efficient control, but in temporary situations, it is possible to take advantage of the redundancy of CVs in group 2. Suitable criticality matrices were defined to correctly define the control matrix to be exploited at each control instant and to deduce whether the cooler APC system could remain active or not.

**Figure 8:**
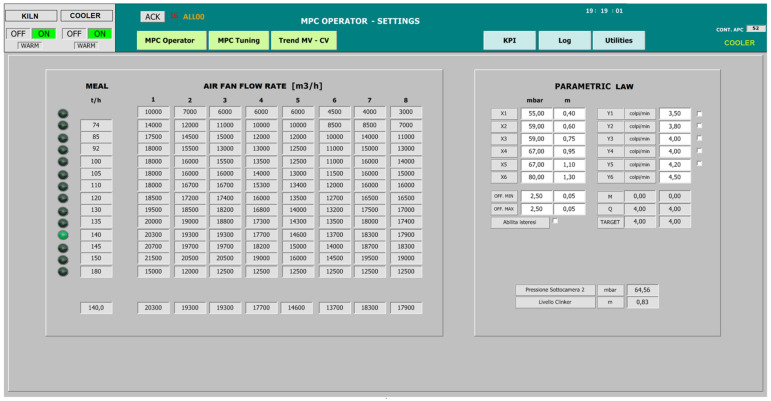
Grate cooler APC system: GUI synoptic for MVs nominal targets computation and parametric law modification.

In order to guarantee the desired control of the clinker exit temperature (cooler CV1, see Table 6), the suitable manipulation of the cold air fan flow rates has to be ensured. For this purpose, nominal targets for the cold air fan flow rates are computed based on the meal flow rate data. These targets are provided by the *SCADA and Database* module to the *MPC* module as external targets (see Figure 5), which are exploited by the *TOCS* block (see $u_{t}{}_{TOCS}$ in Equation (6)) in its QP formulation. Figure 8 shows a synoptic of the GUI designed on the SCADA system; in this synoptic, an example of the nominal targets associated with each meal flow rate range is reported. All the cold air fan flow rates should ideally follow the computed nominal target and not exceed it. For this reason, the nominal targets also define the upper constraints of the fan flow rates in the *TOCS* and *DO* blocks (see $ub_{u\_TOCS}$ term in Equation (7b) and $ub_{u}$ term in Equation (5b)). This setting allows us to limit the electric energy consumption of the cold air fan units. As a tuning choice, the main control objective of MV1-MV5 (see Table 4) is to track their nominal target, while the MV6-MV8 tuning weights in (6) were set to primarily ensure the control of the clinker exit temperature. Consequently, the flow rates of the first five fans (i.e., fans closest to the kiln exit) are moved based on the raw meal introduced into the kiln (their lower and upper constraints were set equal to the same value in the *TOCS* and *DO* formulations). In Equations (4) and (6), the tuning parameters were set to prefer a deviation of the fans more distant from the kiln exit, in order to decrease the air flow rate in the case of a violation of the lower constraint of the clinker exit temperature. In order to allow a decrease in the cold air fan flow rates in this case, a fixed offset was established between the upper and the lower constraints of each fan. The clinker exit temperature (cooler CV1, see Table 6) was characterized only with a lower constraint (see $lb_{y\_TOCS}$ term in Equation (7c) and $lb_{y}$ term in Equation (5c)) because no action is required if the temperature rises.

Since the nominal targets of the fan flow rates are computed based on meal flow rate data, an effective coordination and cooperation is established between the rotary kiln and the grate cooler (see the preliminaries in Section 2.5). The MPC strategy could be effective in anticipating the fan control actions in the grate cooler.

**Figure 9:**
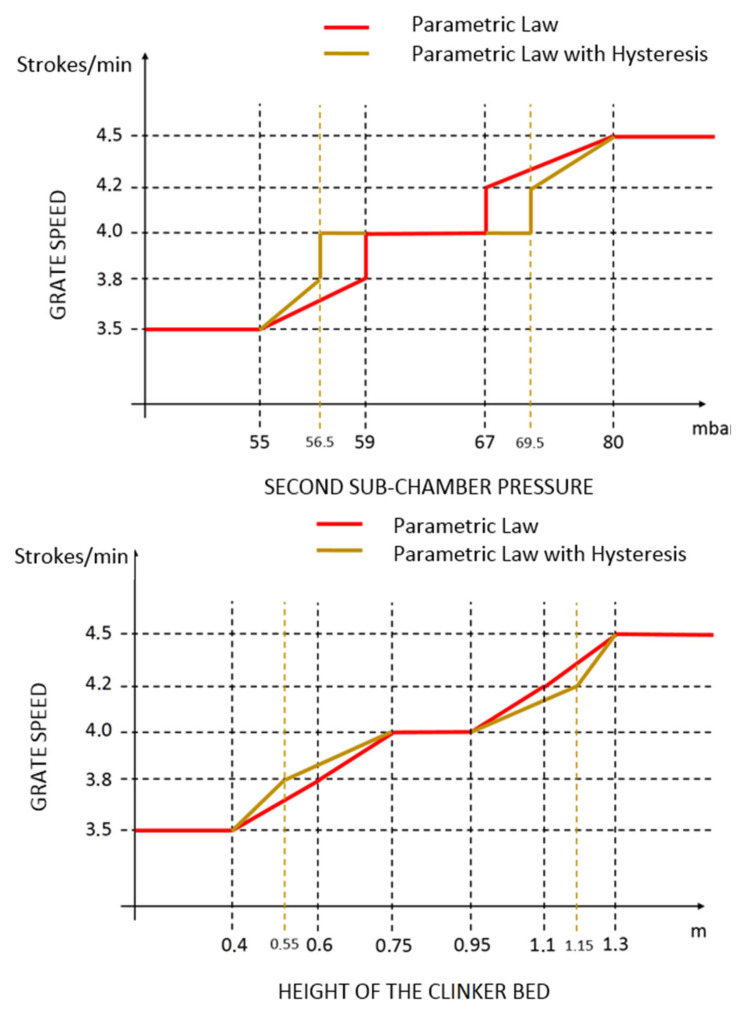
Grate cooler APC system: parametric law between second sub-chamber pressure/height of the clinker bed and grate speed.

In order to control the pressure of the second sub-chamber and the height of the clinker bed (cooler CV2 and CV3, see Table 6), grate speed MV is used. Assuming that both CVs are active, a priority logic is exploited in order to compute the grate speed desired target (see the selector column in Figure 7). The proposed priority logic includes a parametric law (see Figure 9) that is used for the computation of an external target for the grate speed. The parametric law can be modified in real-time (see the right side of Figure 8). A hysteresis is introduced into the parametric law to enable the strokes of the grate cooler to be reset more quickly to a nominal value when the controlled variables were at their nominal values. This allows us to avoid oscillations and, at the same time, to stabilize the grate speed. In Figure 9, the nominal ranges are depicted by the middle horizontal lines. The selector (see Figure 7) allows us to set the higher priority among CV2 and CV3. In this way, when the higher priority CV is within its nominal range, the grate speed is used for the lower priority CV if this CV is outside the nominal range.

To the best of the authors’ knowledge, the proposed method for the exploitation of the sensor redundancy (associated with some CVs) represents a scientific contribution to the literature of grate cooler APC systems. This feature allows us to ensure the best control actions in all operating conditions and also to allow the use of the APC system in the case of unexpected events (e.g., malfunction of a device). In addition, to the best of authors’ knowledge, the overall proposed control method for grate coolers is a scientific contribution to the literature of grate cooler APC systems.

### 2.6. Software

The computational framework exploited for all the project phases, excluding the commissioning phase, was represented by a laptop computer with the following specifications: Intel(R) Core(TM) i8-3840QM CPU with 3 GHz HDD. A MATLAB environment was used for data analysis, modelization and virtual environment simulations. The MATLAB Identification Toolbox, MATLAB Control System Toolbox and MATLAB Optimization Toolbox were exploited for process identification and controller synthesis. Furthermore, a MATLAB environment was also used for the project maintenance in order to analyze APC system performance and KPIs [37]. For the commissioning phase (field implementation), the architecture reported in Figure 8, enriched with the schematic representation of Figure 9, was exploited.

## 3. Results

The project phases reported in Figure 2 were executed in order to target the commissioning of APC systems on the real plant. The commissioning was executed through different steps. Industry 4.0 certification was obtained for the developed APC systems; the architecture depicted in Figure 3 played a key role for this purpose. In the following, some results related to data analysis, modelization, virtual environment simulations, field control performances and KPI evaluation are reported.

### 3.1. Data Analysis and Modelization Results

**Figure 10:**
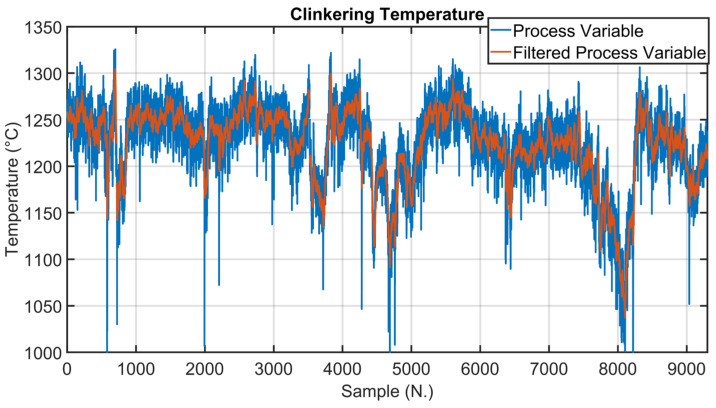
Rotary kiln APC system: data analysis results (clinkering temperature).

**Figure 11:**
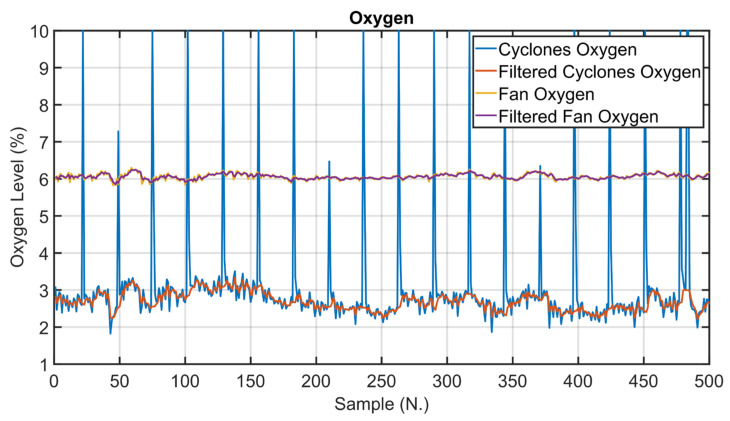
Rotary kiln APC system: data analysis results (oxygen).

**Figure 12:**
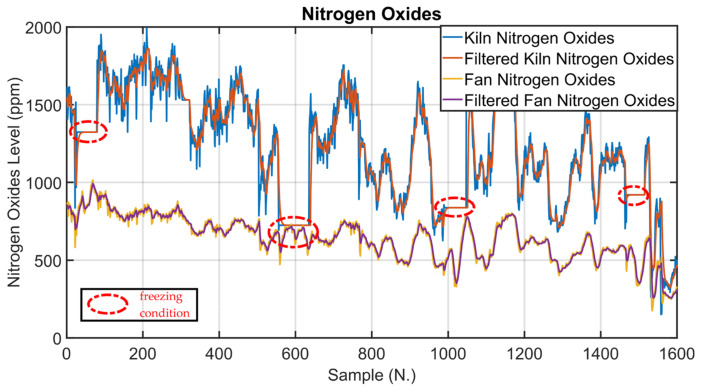
Rotary kiln APC system: data analysis results (nitrogen oxides).

As explained in Section 2.3, data analysis represented one of the key phases of the presented work. In the following, examples of data analysis results for three critical process variables are reported. The process variables taken into account are clinkering temperature, oxygen levels and kiln nitrogen oxides levels (see Figure 1 and Table 3). Clinkering temperature is a significant CV of the kiln APC system because its behavior indicates the progress of the baking of the raw meal within the rotary kiln during its transformation into clinker. As can be observed in Figure 10, this CV (blue line) is characterized by significant oscillations due to the difficulty of its measurement process. For this reason, Equation (1a,b) were exploited for the preprocessing of the clinkering temperature measurement; the δ  parameter was set equal to 1/3. The filtered clinkering temperature is reported in Figure 10 (orange line), and it is used by the *MPC* module (see Figure 5). With regard to oxygen levels, the main oxygen level to be kept under control is represented by the cyclone oxygen; as mentioned in Section 2.3, the measurement of cyclone oxygen can be subjected to drifts in some situations (e.g., cyclone cleaning). This condition is reported in Figure 11, where the process variable (blue line) and the filtered process variable (orange line) of the cyclone oxygen are depicted. In the situations described, as reported in Section 2.5.2, the oxygen to be kept under control is no longer the cyclone oxygen, which is replaced by the fan oxygen since during cyclone cleaning the fan oxygen measurement remains reliable (see Figure 11, yellow line). The purple line in Figure 11 shows the filtered fan oxygen variable. For the computation of the filtered oxygen levels depicted in Figure 11, the δ parameter was set equal to 2/3 (Equation (1a,b)). With regard to nitrogen oxides levels, the key variable is the kiln nitrogen oxides (see Section 2.3). This variable is reported in Figure 12 (blue line), together with its filtered values (orange line). As can be observed, the measurement of kiln nitrogen oxides can be subjected to freezing conditions at different periods (see the red circles). In these cases, the kiln nitrogen oxides is replaced by another CV. In the reported example it is replaced by the fan nitrogen oxides, as reported in Section 2.5.2 (see the yellow and purple lines in Figure 12). The nitrogen oxides filtered values depicted in Figure 12 were obtained by applying Equation (1a,b) with a δ parameter equal to 2/3.

The examples given are significant because they show experimental evidence of the value of sensor redundancy in the cement industry and how data analysis can be exploited to detect process conditions or bad data.

**Figure 13:**
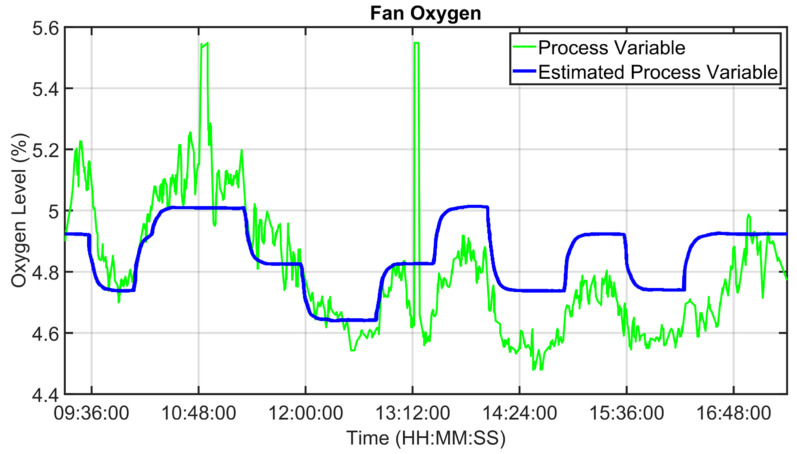
Rotary kiln APC system: modelization results (fan oxygen).

**Figure 14:**
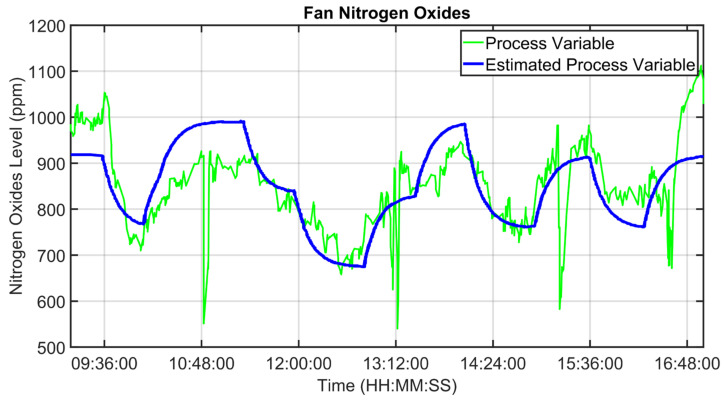
Rotary kiln APC system: modelization results (fan nitrogen oxides).

**Figure 15:**
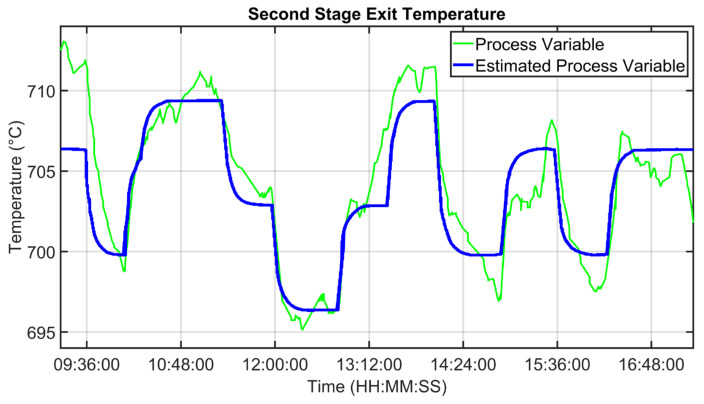
Rotary kiln APC system: modelization results (second-stage exit temperature).

For the MPC design, as reported in Section 2.4, a suitable process modelization was performed. Some modelization results are shown in Figure 13, Figure 14 and Figure 15. Fan oxygen, fan nitrogen oxides and second-stage exit temperature CVs (see Table 3) are considered. The CVs models are validated in an eight-hour period; process variables are depicted through green lines, while blue lines represent the estimation provided by the model. Figure 13 reports fan oxygen model performances, while Figure 14 refers to fan nitrogen oxides. Finally, Figure 15 includes second-stage exit temperature model results. Observing the reported results, it can be noticed how the obtained models allow for an acceptable approximation of the behavior of the considered CVs.

### 3.2. Virtual Environment Simulations Results

**Figure 16:**
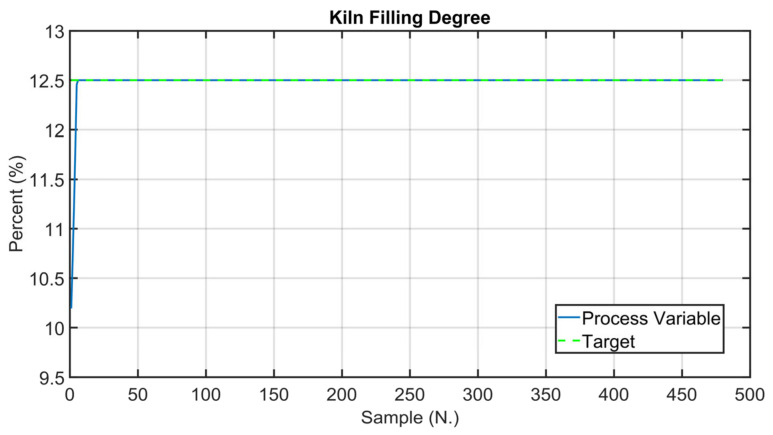
Rotary kiln APC system: virtual environment simulations results (kiln filling degree CV).

**Figure 17:**
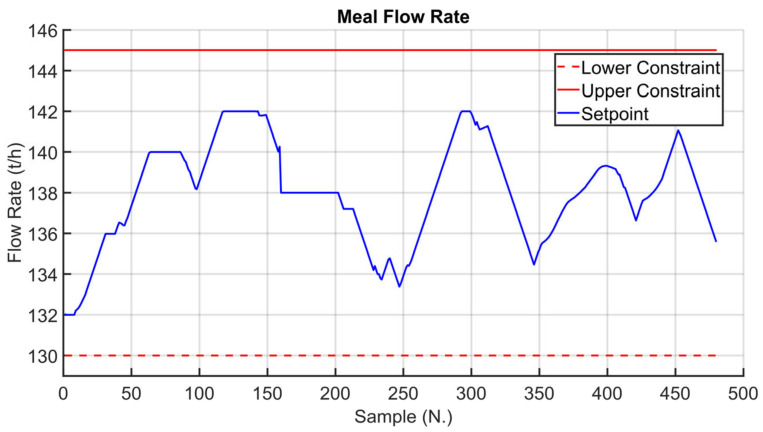
Rotary kiln APC system: virtual environment simulations results (meal flow rate MV).

**Figure 18:**
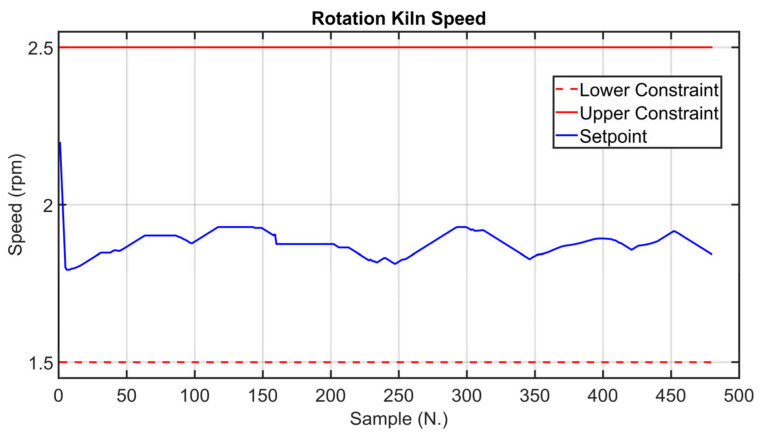
Rotary kiln APC system: virtual environment simulations results (rotation kiln speed MV).

Before the installation of the APC systems on the real plant, tailored virtual environment simulations were performed in order to test the developed controllers. The kiln filling degree CV is considered in the following simulation in order to show the effectiveness of the proposed approach, which exploits the fictitious variable z (see Equation (3)). The results in Figure 16, Figure 17 and Figure 18 refer to an eight-hour-period simulation. As reported in Section 2.4 and in Section 2.5, this CV is controlled using Equation (3) in the *MPC* module. The kiln filling degree CV is reported in Figure 16 (blue line), together with its target (green dotted line). The target corresponds to the term $CV13_{t}\left(k\right)$ of Equation (3). As reported in Equation (3), the kiln filling degree depends on the meal flow rate and on the rotation kiln speed MVs, but only the rotation kiln speed MV can be used for its control (see Section 2.1). In general, the meal flow rate is moved by the *MPC* module in order to control the other active CVs and to pursue the mentioned economic objectives (see Section 2.5). In these situations, as a consequence, the rotation kiln speed must be manipulated by the *MPC* module in order to satisfy the kiln filling degree control specification. The meal flow rate setpoint is reported in Figure 17 (blue line), together with its constraints (red lines). Taking into account meal flow rate values, the rotation kiln speed setpoint (see Figure 18, blue line) is exploited by the *MPC* module within the defined constraints (see Figure 18, red lines) in order to reach the target associated with the kiln filling degree. At the beginning of the simulation, the kiln APC system is not active, and the kiln filling degree is not respecting the assigned target (see the left side of Figure 16). The kiln APC system is switched on and requires a decrease of the rotation kiln speed (see Figure 18) in order to allow target tracking by the kiln filling degree. After a brief transient period, due to the fact that rotation kiln speed is subjected to *hard* move constraints (see Section 2.5) with an absolute value equal to 0.1 rpm, the kiln filling degree reaches the desired value (see Figure 16) and remains at this value despite meal flow rate variations (see Figure 17).

### 3.3. Field APC Results

Examples of field performances of the developed APC systems are reported and discussed in the following.

**Figure 19:**
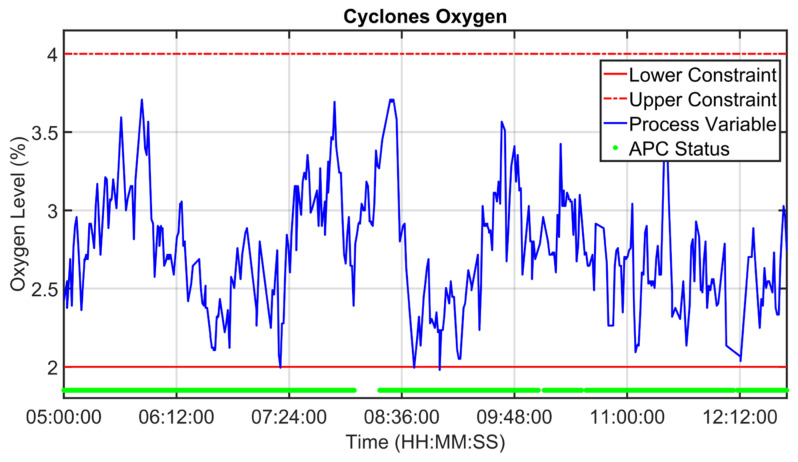
Rotary kiln APC system: field results (cyclone oxygen CV).

**Figure 20:**
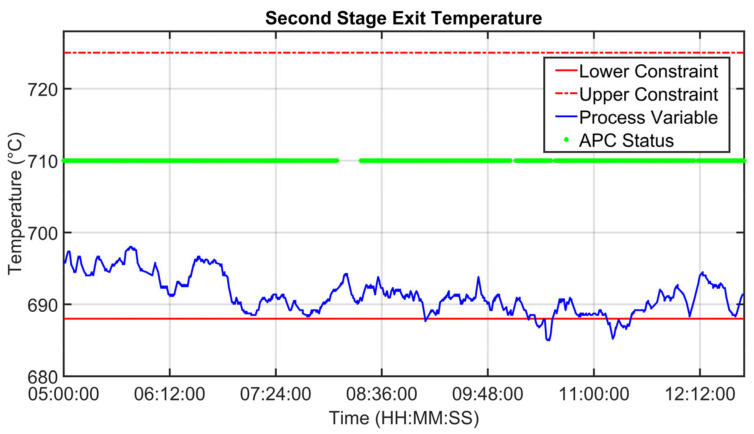
Rotary kiln APC system: field results (second-stage exit temperature CV).

**Figure 21:**
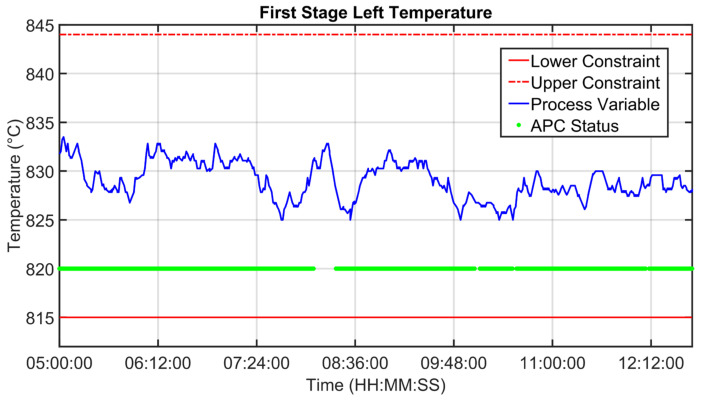
Rotary kiln APC system: field results (first-stage left temperature CV).

**Figure 22:**
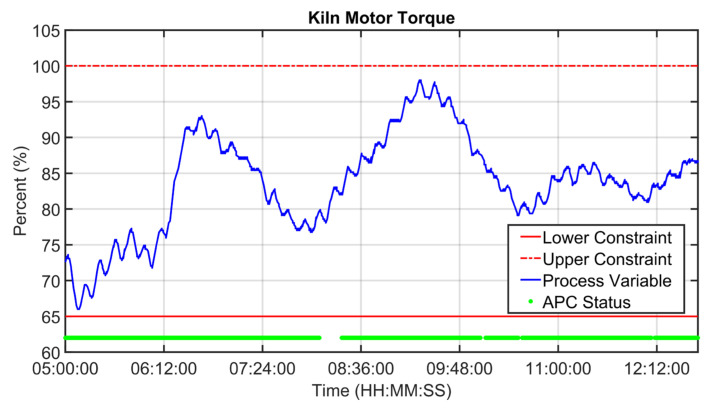
Rotary kiln APC system: field results (kiln motor torque CV).

**Figure 23:**
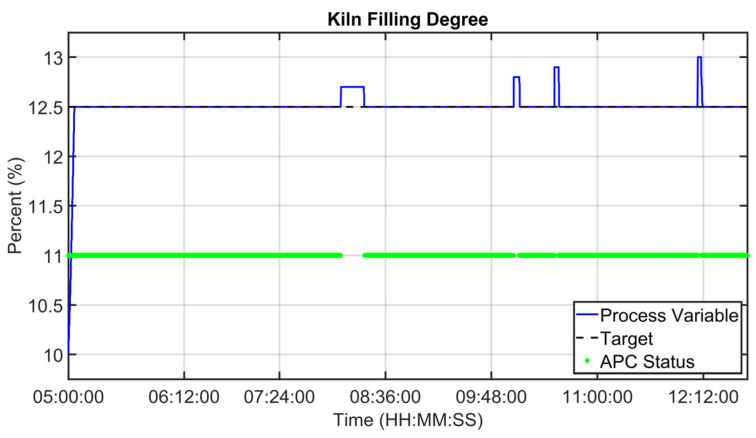
Rotary kiln APC system: field results (kiln filling degree CV).

**Figure 24:**
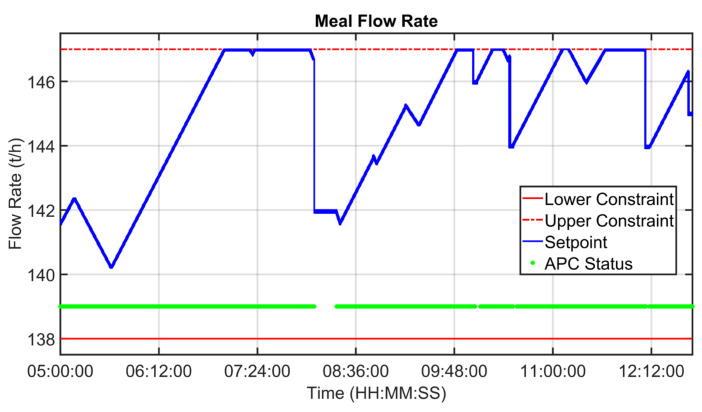
Rotary kiln APC system: field results (meal flow rate MV).

**Figure 25:**
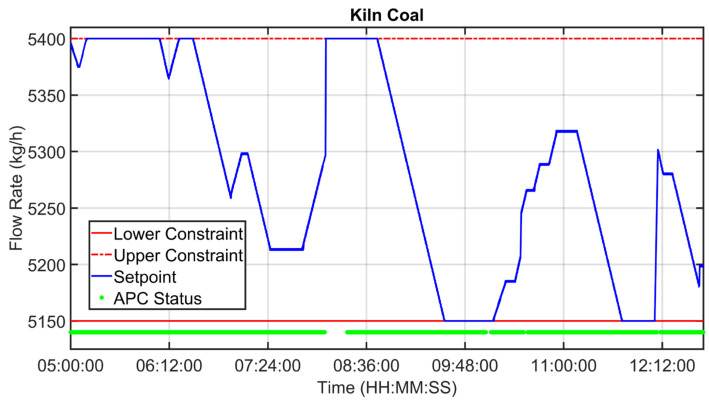
Rotary kiln APC system: field results (kiln coal MV).

**Figure 26:**
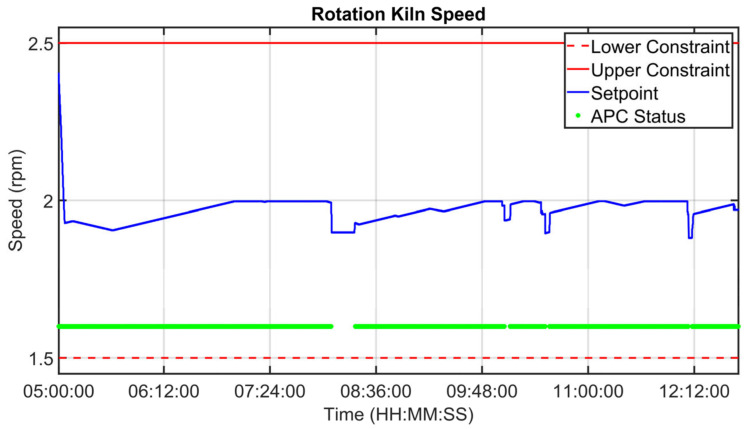
Rotary kiln APC system: field results (rotation kiln speed MV).

Figure 19, Figure 20, Figure 21, Figure 22, Figure 23, Figure 24, Figure 25 and Figure 26 report an example of control performances provided by the kiln APC system over a period of about eight hours. Figure 19, Figure 20, Figure 21, Figure 22 and Figure 23 depict some CVs, i.e., cyclone oxygen, second-stage exit temperature, first-stage left temperature, kiln motor torque and kiln filling degree. In these figures, the constraints (where present) are shown through red lines, the targets (where present) are shown through black lines, the process variables are depicted through blue lines, and the supervised APC status flag (see Section 2.5) is represented by green lines. Figure 24, Figure 25 and Figure 26 depict some MVs (meal flow rate, kiln coal and rotation kiln speed). In these figures, the constraints are shown through red lines, setpoints are depicted through blue lines, and the supervised APC status flag (see Section 2.5) is represented by green lines. In the first part of the selected period, the kiln APC system is switched on, and it controls and optimizes the process: the APC system maintains the CVs within their constraints, suitably moving the MVs. In addition, the target imposed on the kiln filling degree is achieved after a brief transient period, confirming the field reliability of the virtual environment simulation shown in Section 3.2. It can be observed how meal flow rate is moved toward its upper constraint in order to ensure the decreasing of the fuel/coal specific consumption. Observing the green lines in Figure 19, Figure 20, Figure 21, Figure 22, Figure 23, Figure 24, Figure 25 and Figure 26, it can be noticed that sometimes the kiln APC system is switched off due to temporary process maintenance operations, which require conservative values for the meal flow rate and the kiln coal MVs (see Figure 23 and Figure 24). In addition, the plant operators also decrease the rotation kiln speed MV SP (see Figure 25) in order to maintain the desired target for the kiln filling degree CV (see Figure 23). As can be noted in Figure 23, the kiln filling degree target is not ensured when the APC system is not active. When the APC system is switched on again, it moves the MVs in order to ensure the CVs constraints and targets tightening while searching for optimal values that minimize the specific consumption. In particular, in the second part of the plots, the APC system minimizes the coal and maximizes the meal flow rate. All the constraint violation magnitudes (see for example the second-stage exit temperature in Figure 20) are negligible.

**Figure 27:**
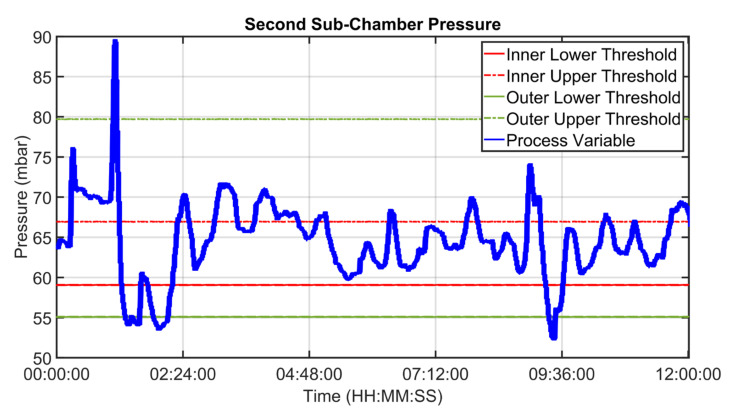
Grate cooler APC system: field results (second sub-chamber pressure CV).

**Figure 28:**
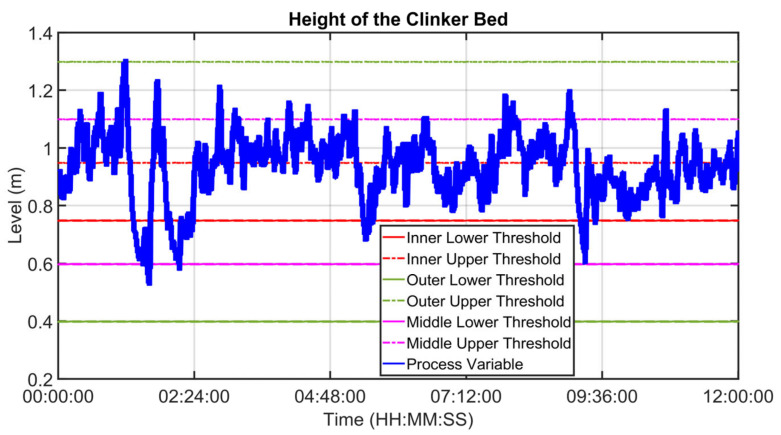
Grate cooler APC system: field results (height of the clinker bed CV).

**Figure 29:**
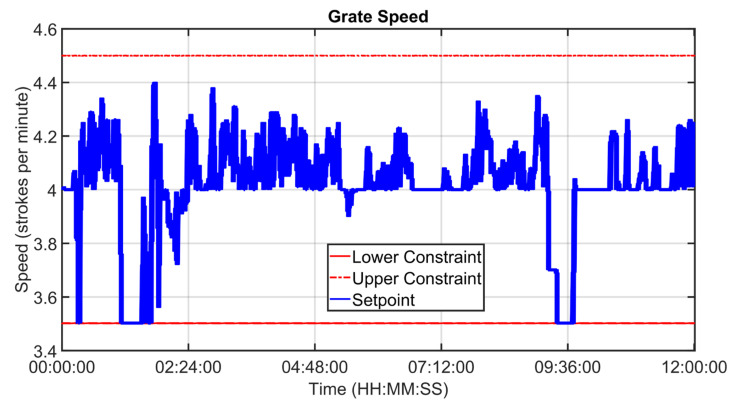
Grate cooler APC system: field results (grate speed MV).

**Figure 30:**
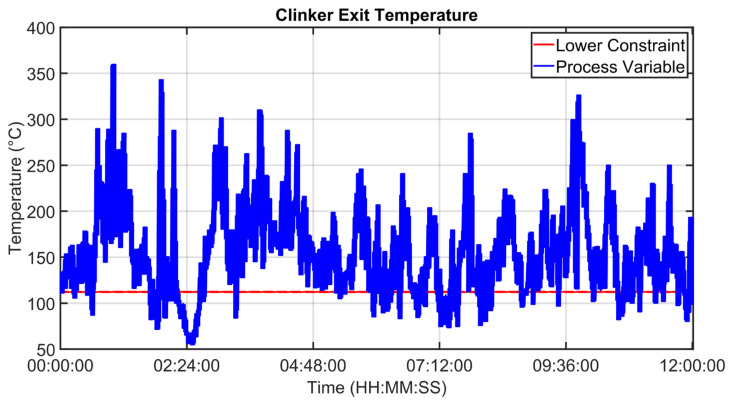
Grate cooler APC system: field results (clinker exit temperature CV).

**Figure 31:**
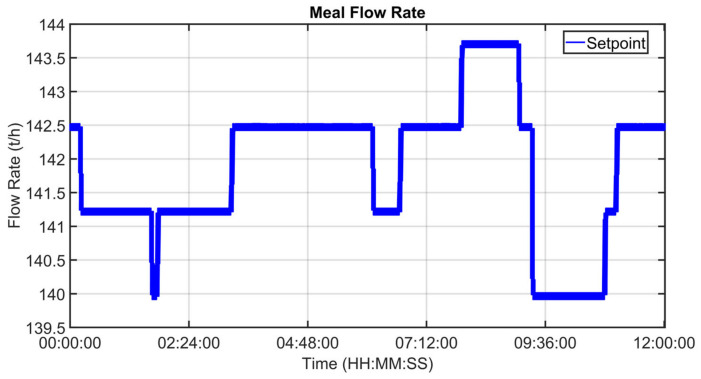
Grate cooler APC system: field results (meal flow rate DV).

**Figure 32:**
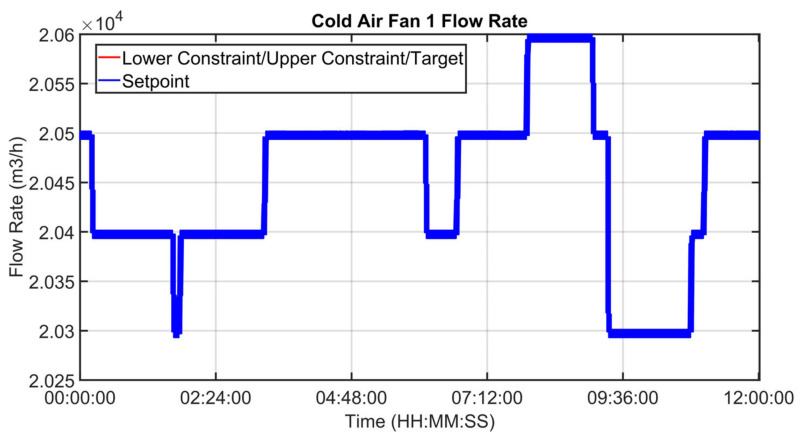
Grate cooler APC system: field results (cold air fan 1 flow rate MV).

**Figure 33:**
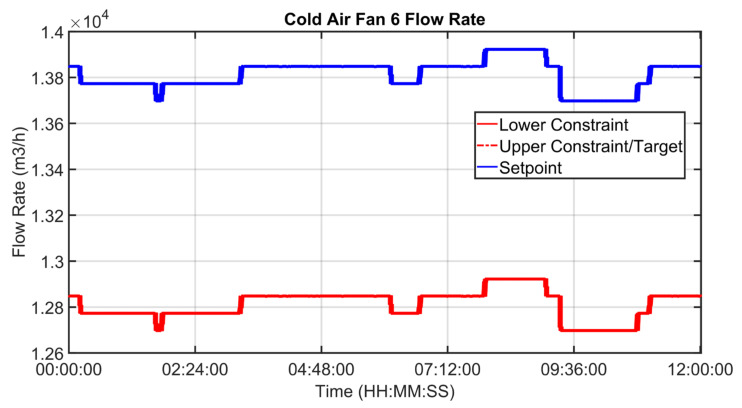
Grate cooler APC system: field results (cold air fan 6 flow rate MV).

**Figure 34:**
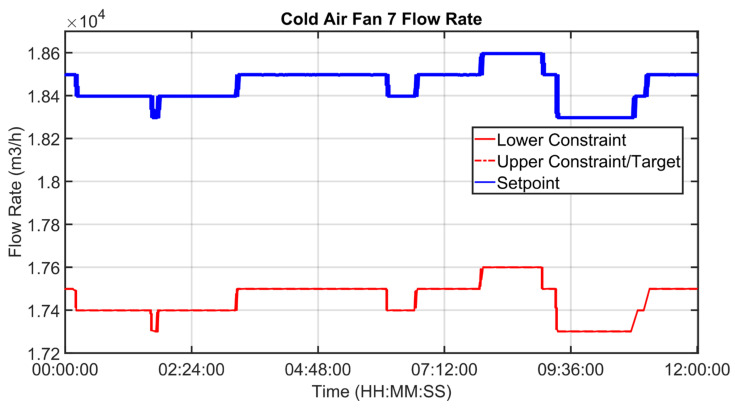
Grate cooler APC system: field results (cold air fan 7 flow rate MV).

**Figure 35:**
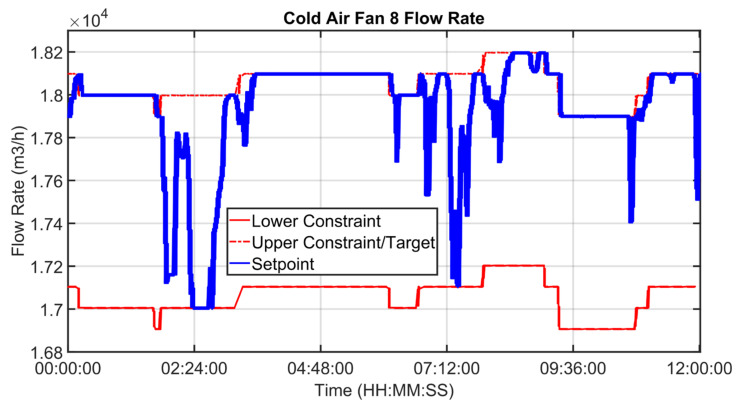
Grate cooler APC system: field results (cold air fan 8 flow rate MV).

An example of control performances provided by the cooler APC system over a period of about twelve hours is reported in Figure 27, Figure 28, Figure 29, Figure 30, Figure 31, Figure 32, Figure 33, Figure 34 and Figure 35. The APC status flag is not reported because in the considered period the controller is always active. Figure 27, Figure 28 and Figure 29 show how the grate speed MV (Figure 29) is exploited by the APC system to control the two associated CVs, i.e., the second sub-chamber pressure (Figure 27) and the height of the clinker bed (Figure 28). In Figure 27 and Figure 28, the CVs process variables are represented through blue lines, while the thresholds of the parametric law used for the grate speed MV target computation (see Section 2.5) are depicted through red, magenta and green lines. In Figure 29, the grate speed MV setpoint is depicted through a blue line, while the associated magnitude constraints are depicted through red lines.

In the reported example, the height of the clinker bed CV has a higher priority with respect to the second sub-chamber pressure, i.e., the selector of Figure 7 is switched to the height of the clinker bed. Thanks to the developed control strategy, the APC system is able to guarantee an acceptable control of the considered CVs, suitably using the MV. The moves computed by the controller are targeted to respect the defined thresholds for the CVs and are scaled on the computed violation of the defined thresholds.

Figure 30, Figure 31, Figure 32, Figure 33, Figure 34 and Figure 35 show how some cold air fan units are handled by the cooler APC system in order to control the clinker exit temperature CV, taking into account the raw meal that is fed to the kiln. The clinker exit temperature CV is reported in Figure 30: the blue line depicts the process variable, while the defined lower constraint is illustrated through a red line. The meal flow rate (see Figure 31) is considered as a DV for the cooler APC system (see Table 5). Figure 32, Figure 33, Figure 34 and Figure 35 report some cold air fan unit flow rate MVs: the setpoint is depicted through blue lines, while the constraints and targets are represented by red lines. With regard to the first five fan units, only the first one is reported for brevity due to the fact that their behavior is very similar. With regard to the first five fan units, the target defines also the upper and lower constraints (see Figure 32 for the first fan unit); on the other hand, the last three fan units, i.e., the fan units located further away from the kiln outlet, are equipped with a nominal target that must not be exceeded. For this reason, the nominal target of the flow rates of the last three fan units overlaps the upper constraint in Figure 33, Figure 34 and Figure 35. In addition, an offset equal to 1000 m³/h is applied to compute the cold air flow rate lower constraint of each of the last three fan units (see Figure 33, Figure 34 and Figure 35), as described in Section 2.5. The provided rangeability for the last three fan units allows the APC system to decrease the cold air flow rate setpoint in the case of a reduction in the clinker exit temperature. As can be noted in Figure 32, Figure 33, Figure 34 and Figure 35, the variation of the constraints and targets related to the MVs corresponds to a change in the raw meal that enters the kiln. In this way, a coordination between the control of the two subprocesses (rotary kiln and grate cooler) is obtained. Furthermore, when the clinker exit temperature approaches its minimum constraint (see Figure 30), the cooler APC system decreases the flow rate of the last fan unit (MV8, see Figure 35), while leaving the previous ones to their nominal targets (see, for example, MV7 in Figure 34).

By installing the cooler APC system, the automatic conduction of the cooler fans flow rate has been obtained, according to the meal flow rate of the rotary kiln. In this way, abnormal conditions related to the air (excess or excessive reduction) are avoided, which could cause dangerous disturbances in the combustion of the kiln. For example, the risk of raising dust from the grate cooler (thus causing problems of occlusion of the air ducts) and of lowering the clinker exit temperature are conditions that compromise the quality of the final product. In fact, the air recovered from the grate cooler—strongly influenced by the clinker distribution in it—is used as the secondary air and tertiary air of the kiln (see Section 2.1).

### 3.4. KPI Evaluation

As reported in Section 2.1 (see Figure 2), KPIs were evaluated after the commissioning phase. The selection of the KPIs was performed thanks to the accurate data analysis phase (see Section 2.3), where tailored indicators were defined for the rotary kiln and for the grate cooler.

**Figure 36:**
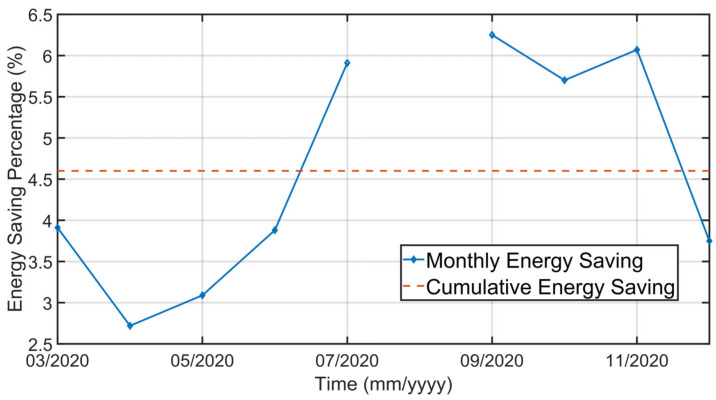
Rotary kiln APC system: KPI evaluation (monthly energy-saving and cumulative energy-saving after APC system activation).

With regard to the rotary kiln, different procedures were performed for the KPI evaluation (see Section 2.3). A comparison between the previous control system and the developed kiln APC system related to the cyclone oxygen percentage and kiln nitrogen oxides (ppm) control was executed, taking into account the mean value and the standard deviation; the comparison was performed through a performance test and a total period of about three weeks was considered. Thanks to the optimized management of the MVs used for their control, a reduction in their standard deviation is observed. In particular, about a 39% reduction was registered for the standard deviation of cyclone oxygen, together with about a 3% increase on its mean value. Furthermore, about a 32% reduction was registered for the standard deviation of kiln nitrogen oxides, together with about a 15% decrease in its mean value. This result is very meaningful, because it implies a significant emissions reduction, thus allowing for a decrease in environmental impact. The activation of the kiln APC system reduced the kiln fuel/coal average specific consumption, as well. Figure 36 shows the results obtained during the period March-December 2020. The blue line indicates the monthly energy-saving with respect to the computed baseline, while the red line reports the cumulative energy-saving (about a 4.6% saving). In Figure 36, January, February and August data are not present, due to maintenance operations on the plant. The average specific consumption reduction and, more generally, the energy efficiency achievement (that also involves emissions reduction) allowed us to obtain Italian energy efficiency certificates (Italian acronym TEE).

With regard to the grate cooler, different procedures were performed for the KPI evaluation (see Section 2.3). First, some statistical properties of the kiln tertiary air temperature and of the second sub-chamber pressure were computed, taking into account a period of three months before and after the commissioning of the cooler APC system. As mentioned in Section 2.3, it was not possible to use as a KPI the height of the clinker bed because the sensor was installed after the commissioning of the cooler APC system. Second, a tailored KPI was introduced: a counter related to the second sub-chamber pressure, which measures the times that this process variable exceeds a maximum pressure threshold (equal to 73 mbar). Table 11, Table 12 and Table 13 report the obtained results. After the installation of the designed APC system, a 4.7% increase of the temperature related to the kiln tertiary air was obtained (see Table 11). In this way, a satisfactory contribution to the kiln combustion process was registered. Furthermore, a remarkable reduction in the standard deviation related to the second sub-chamber pressure was observed (see Table 12), together with a reduction in the violation of the defined maximum pressure threshold (see Table 13). In addition, the grate cooler APC system achieved a mean yearly saving equal to about 10% for electric energy consumption. Finally, a service factor greater than 94% was obtained after more than two years after commissioning the grate cooler APC.

Finally, the reported qualitative and quantitative analysis of the KPIs proves the robustness and reliability of the proposed APC systems.

## 4. Conclusions

In this paper, the design and the field implementation of APC systems for the clinker rotary kiln and grate cooler of a cement industry were proposed. MPC was exploited as a control strategy; in particular, a tailored two-layer MPC scheme was proposed. Different technical and methodological novelties were provided with regard to the literature of APC systems for clinker rotary kilns and grate coolers. The innovative methods concerned:

- The procedural steps for a qualified plant survey;
- An in-depth customization of the data selection, acquisition, storage and analysis phases;
- An overall assessment of the KPIs to be computed and exploited for the evaluation of the performance, provided by an APC system on a grate cooler;
- The definition of the control matrix;
- The MPC problem formulation and tuning;
- A feature to take into account bad data detection and local control loops malfunctions in the APC systems architecture;
- The exploitation of the sensor/analyzer redundancy in an APC system;
- A cooperation policy between the rotary kiln and the grate cooler APC systems.

Virtual environment simulations allowed us to test and check all the designed features within the APC systems. The overall control system was installed on the real plant, obtaining significant results in terms of service factor and control and energy-saving performance. With regard to the rotary kiln, significant improvements in the control of the critical process variables, e.g., oxygens and nitrogen oxides level, were observed after the installation on the real plant. The consequent reduction in their standard deviation allowed for a safe approach to process operating limits, leading to fuel/coal specific consumption reduction and the achievement of energy efficiency certificates. With regard to the grate cooler, the APC system increased the average tertiary air temperature and synergistically contributed to the combustion efficiency in the kiln. This improvement was possible due to the optimization of the conduction of the grate cooler, based on the grate speed regulation and on the automation of the regulation of the fans in the sub-chambers, which takes into account the raw meal flow rate. In addition, a decrease in the electrical energy consumption of the cold air fan units was obtained while ensuring the recovery of the needed heat to the kiln and to the preheater.

Future work will be focused on the extension of the developed control systems to other significant subprocesses of the cement industry, e.g., the mill. In addition, further theoretical and practical improvements for the developed methods will be investigated.

## Figures and Tables

**Table 1:** Rotary kiln APC system: MVs.

Variable Name	Range Example
Meal Flow Rate	130–140 [t/h]
Kiln Coal	3900–4500 [kg/h]
Rotation Kiln Speed	1–2.5 [rpm]
ID Fan Speed	660–690 [rpm]

**Table 2:** Rotary kiln APC system: DVs.

Variable Name	Range Example
Precalciner Coal	3800–4100 [kg/h]
Solid Secondary Fuel	0–3 [t/h]
Kiln Tertiary Air	4–40 [%]
Precalciner Tertiary Air	10–50 [%]
Radial Air Pressure	100–250 [mbar]

**Table 3:** Rotary kiln APC system: main CVs.

Variable Name	Range Example
Cyclone Oxygen	1.5–3 [%]
Fan Oxygen	5.3–7 [%]
Kiln Nitrogen Oxides	1100–1230 [ppm]
Fan Nitrogen Oxides	700–800 [ppm]
Cyclone Carbon Monoxide	0–0.06 [%]
First-Stage Right Temperature	830–860 [°C]
First-Stage Left Temperature	830–860 [°C]
Second-Stage Exit Temperature	705–730 [°C]
Kiln Motor Power	105–125 [kW]
Kiln Motor Torque	20–40 [%]
Clinkering Temperature	1100–1300 [°C]
Chimney Ammonia Flow Rate	240–280 [t/h]
Kiln Filling Degree	10–20 [%]

**Table 4:** Grate cooler APC system: MVs.

Variable Name	Range Example
Cold Air Fan 1 Flow Rate	10,000–21,500 [m^3^/h]
Cold Air Fan 2 Flow Rate	7000–20,500 [m^3^/h]
Cold Air Fan 3 Flow Rate	6000–20,500 [m^3^/h]
Cold Air Fan 4 Flow Rate	6000–19,000 [m^3^/h]
Cold Air Fan 5 Flow Rate	6000–16,000 [m^3^/h]
Cold Air Fan 6 Flow Rate	4500–14,500 [m^3^/h]
Cold Air Fan 7 Flow Rate	4000–19,500 [m^3^/h]
Cold Air Fan 8 Flow Rate	3000–19,000 [m^3^/h]
Grate Speed	3.5–4.5 strokes per minute

**Table 5:** Grate cooler APC system: DVs.

Variable Name	Range Example
Meal Flow Rate	130–140 [t/h]

**Table 6:** Grate cooler APC system: CVs.

Variable Name	Range Example
Clinker Exit Temperature	50–350 [°C]
Second Sub-Chamber Pressure	55–80 [mbar]
Height of the Clinker Bed	0.4–1.3 [m]

**Table 7:** Rotary kiln APC system: main CVs grouping policy.

Variable Name	Group
Cyclone Oxygen	1
Fan Oxygen	1
Kiln Nitrogen Oxides	2
Fan Nitrogen Oxides	2
Cyclone Carbon Monoxide	3
First-Stage Right Temperature	4
First-Stage Left Temperature	4
Second-Stage Exit Temperature	4
Kiln Motor Power	5
Kiln Motor Torque	5
Clinkering Temperature	6
Chimney Ammonia Flow Rate	2
Kiln Filling Degree	7

**Table 8:** Rotary kiln APC system: initial decoupling matrix.

Variable Name	Meal Flow Rate	Kiln Coal	Kiln Speed	ID Fan Speed
Cyclone Oxygen	1	1	1	1
Fan Oxygen	1	1	1	1
Kiln Nitrogen Oxides	1	1	1	0
Fan Nitrogen Oxides	1	1	1	1
Cyclone Carbon Monoxide	1	1	1	1
First-Stage Right Temp.	1	1	1	0
First-Stage Left Temp.	1	1	1	0
Second-Stage Exit Temp.	1	0	1	0
Kiln Motor Power	1	1	0	1
Kiln Motor Torque	1	1	0	1
Clinkering Temperature	1	1	1	0
Chimney Amm. Flow Rate	1	1	1	1
Kiln Filling Degree	0	1	1	1

**Table 9:** Rotary kiln APC system: priority rankings for constraints relaxation.

CVs Group	Ranking 1	Ranking 2
1	1	1
2	3	1
3	6	5
4	2	2
5	4	4
6	5	3
7	1	1

**Table 10:** Grate cooler APC system: main CVs grouping policy.

Variable Name	Group
Clinker Exit Temperature	1
Second Sub-Chamber Pressure	2
Height of the Clinker Bed	2

**Table 11:** Grate cooler APC system: KPI evaluation (kiln tertiary air temperature).

Kiln Tertiary Air Temperature				
	Mean Value		Standard Deviation	
**Before**	891.88 [°C]		25.61 [°C]	
**After**	935.18 [°C]	+4.7 [%]	30.54 [°C]	+19.3 [%]

**Table 12:** Grate cooler APC system: KPI evaluation (second sub-chamber pressure).

Second Sub-Chamber Pressure				
	Mean Value		Standard Deviation	
**Before**	65.44 [mbar]		3.25 [mbar]	
**After**	62.87 [mbar]	−3.9 [%]	2.76 [mbar]	−15.2 [%]

**Table 13:** Grate cooler APC system: KPI evaluation (second sub-chamber pressure counter).

Second Sub-Chamber Pressure		
	≥73 [mbar]	
**Before**	3.44 [%]	
**After**	0.38 [%]	−89 [%]

## Data Availability

Not applicable.
